# Emerging therapies for breast cancer

**DOI:** 10.1186/s13045-017-0466-3

**Published:** 2017-04-28

**Authors:** Xichun Hu, Wei Huang, Minhao Fan

**Affiliations:** 1 Department of Medical Oncology, Fudan University Shanghai Cancer Center, Shanghai, 200032 China; 2 Department of Oncology, Shanghai Medical College, Fudan University, Shanghai, 200032 China; 3 Roche Product Development in Asia Pacific.5F, Tower C, Parkview Green, No.9, Dongdaqiao Road, Chaoyang District, Beijing, 100020 People’s Republic of China; 4 Hutchison MediPharma Limited, Building 4 917 Halei Road Zhangjiang Hi-Tech Park, Shanghai, 201203 China

**Keywords:** Breast cancer, CDK4/6 inhibitors, SERD, PD1 and PD-L1 antibodies, PARP inhibitors, Fulvestrant, ADC, Nab-paclitaxel

## Abstract

HER2 and CDK4/6 are undoubted two most important biological targets for breast cancer. Anti-HER2 treatments enhance objective response and progression-free survival/disease-free survival as well as overall survival. Three CDK4/6 inhibitors consistently improve objective response and progression-free survival; however, overall survival data are waited. Optimization of chemotherapy and endocrine strategies remains an unmet need. Check point inhibitor-based immunotherapy combined with chemotherapy is a promising field, especially for triple-negative breast cancer.

## Background

The median overall survival of metastatic breast cancer (MBC) is about 2 to 3 years. Although it is still an incurable disease for more than 90% of MBC patients, much progress has been made in the past decade. The longest reported median overall survival was 56.5 months for HER2-positive MBC patients with first-line treatment of docetaxel, trastuzumab, and pertuzumab, while it was 54.1 months for luminal subtype MBC patients with first-line fulvestrant treatment. This review will focus on drug developments in metastatic setting.

## CDK4/6 inhibitors

Estrogens [1] and antiestrogens [2] act on sensitive populations of cells in early to mid-G1 phase of ER-positive breast cancer cell lines. CDK4 and CDK6 are activated by binding to D-type cyclins and act early in G1 phase [3–6]. CDK in G1 phase mainly targets the retinoblastoma susceptibility gene product (pRb), which mediates G1 arrest through sequestration of transcriptional factors of the E2F-DP family and then transcription of requisite genes for S-phase entry [6, 7].

Inhibitors of CDK4 and CDK6 entering clinical trials include palbociclib, ribociclib, and abemaciclib [8]. Palbociclib blocks ATP binding to the CDK4/6 enzymes with half-maximal inhibitory concentration (IC50) 0.01 μmol/L for CDK4/cyclin D1, 0.009 μmol/L for CDK4/cyclin D3, and 0.015 μmol/L for CDK6/cyclin D2 complexes [9]. The combination of palbociclib with endocrine therapy has synergistic effects in ER+ human breast cancer cell lines [10] as well as xenograft models, and in tamoxifen-resistant breast tumor, the synergy is also present between palbociclib and selective estrogen receptor downregulator [11]. Therefore, endocrine therapies and inhibitors targeting CDK4/6 activity are the core treatment modality in patients with HR+ advanced breast cancer [12].

Besides their antiproliferative activity, palbociclib has shown strong antimetastatic activity in a dose-dependent manner through reducing cyclooxygenase-II expression in MDA-MB-231 (ERα−) and T47D (ERα+) breast cancer cells [13]. Cyclooxygenase-II gene is associated with the activation of epithelial-to-mesenchymal transition process, which helps the epithelial cells to lose their epithelial characteristics and gains mesenchymal characteristics, therefore increasing their invasive and metastatic potentials [14]. Additionally, palbociclib-induced CDK4/6 inhibition can lead to senescence of melanoma cell lines via promoting Forkhead Box M1 degradation [15]. Moreover, cyclin D1 is one of the ER transcriptional targets, thus rationalizing the use of CDK4/6 inhibitors in ERα+ breast cancer [16].

Predictive factors for CDK4/6 inhibitors may include cyclin D1and phosphorylated Rb for sensitivity [8, 17] and p16 for resistance. The cyclin D-CDK4/6-INK4-Rb pathway is frequently deregulated in breast cancer via *CCND1* (cyclin D1) amplification (29–58%), *CDK4* (14–25%) and *CDK6* amplification [18], p16 loss (49%) [19], and *TP53* inactivation (12–84%) [18]. Cyclin D1 overexpression and Rb phosphorylation in ERα+ cancers contribute to the drug resistance to the hormonal therapy [20–24]. Abemaciclib effectively induces the G1 cell cycle arrest, which is dependent upon the presence of Rb. p16 overexpression in Rb-deficient breast cancer cells might account for the resistance to palbociclib, as CDK4/6 enzymes might be already inhibited by the overexpressed p16 [25]. Moreover, ERα+ subtype shows the highest sensitivity to CDK inhibitors, possibly due to the hyperactivation of CDK4/6, while palbociclib showed no antiproliferative effect in Rb-deficient MDA-MB-468 (ERα−) human breast cancer cell lines [8, 26–28]. However, the value of any of these biomarkers was not confirmed in translational studies of clinical trials.

## Palbociclib

Phase I studies using single-agent palbociclib 2/1 (2-week on and 1-week off) schedule [29] and 3/1 (3-week on and 1-week off) schedule [30] were done to identify the dose-limiting toxicity (DLT) and maximum tolerated dose (MTD) of the first-in-class, oral CDK4/6 inhibitor in Caucasian and Japanese patients [31]. The MTD of 3/1 schedule was 125 mg once daily and recommended for further development. Palbociclib was well tolerated, and neutropenia was the only significant DLT.

Phase II study of palbociclib used a single agent in advanced breast cancer [32]. Eligible patients had Rb-positive MBC. Of the 37 enrolled patients, 33 patients were HR+ (7% ERα+, 4% PR+, and 22% ERα+/PR+). Clinical benefit rate was 21% for patients with HR+ and 29% for patients with HR+/HER2− who were exposed to at least two prior lines of hormonal therapy. Progression-free survival (PFS) was significantly longer for patients with HR+ rather than HR− (*p* = 0.03). Most adverse events were myelosuppression. Neutropenia (grade 3/4) was common with 46% requiring dose modifications.

The first combination trial is a randomized, multicenter active-controlled phase I/II study (PALOMA-1) designed to assess the efficacy, safety, and pharmacokinetics of letrozole 2.5 mg QD (continuously) in combination with palbociclib 125 mg QD (schedule 3/1) vs single-agent letrozole 2.5 mg QD (continuously) for the first-line treatment of ER(+) and HER2 (−) ABC in postmenopausal women. Phase I results showed no drug–drug interactions between letrozole and palbociclib. The recommended phase II dose is at 125 mg once daily on 3/1 schedule [33]. The phase II portion consisted of two cohorts. In cohort 1, patient selection was based only on ER/HER2 status. In cohort 2, tumor CCND1 amplification and/or p16 loss were eligibility criteria. The final analysis reported results from both cohorts combined (cohort 1, *n* = 66; cohort 2, *n* = 99). At data cutoff for final analysis (November 29, 2013), PFS was significantly improved with palbociclib (20.2 vs 10.2 months), leading to its conditional FDA approval in February 2015 [34]. PALOMA-2 is a confirmative phase III trial. The study met its primary endpoint [35].

In the second- or later-line setting, the value of palbociclib was assessed in the prospective, multicenter, double-blind phase III PALOMA-3 study in pre/peri- and postmenopausal women with hormone receptor-positive/HER2-negative MBC after progression on endocrine therapy. Patients were randomized to receive fulvestrant with either palbociclib or placebo. Palbociclib was associated with longer progression-free survival (PFS; 11.2 vs 4.6 months, HR = 0.497, *p* < 0.0001) [36]. Its efficacy is independent on PIK3CA mutation status. On February 19, 2016, the US Food and Drug Administration (FDA) approved a new indication for palbociclib based on this result [37]. Overall, findings across the studies suggest palbociclib has synergy with endocrine therapy in both endocrine-naive and endocrine-resistant settings.

Ongoing clinical trials include the HR+/HER2+ study (PATINA) exploring in the HER2+ first-line MBC setting (NCT02947685), adjuvant study PALLAS and PENELOPE-B (NCT01864746, NCT02513394), and PALOMA-4, PEARL, PARSIFAL and NCIC MA-38 (PALESTRA) study (NCT02630693, NCT02491983, NCT02028507, and NCT02297438, respectively) in the MBC setting. Future research directions include deep diving of translational markers, mechanisms, and treatment strategies after endocrine or CDK4/6 inhibitor resistance, Asia population.

## Ribociclib

Ribociclib (LEE011) is an orally bioavailable, highly selective CDK4/6 inhibitor which is also in later stages of clinical development. In preclinical studies, ribociclib caused inhibition of tumor growth and cell cycle arrest in several Rb-proficient cell lines and in a dose-dependent manner [38]. The antitumor activity is confirmed in a variety of xenograft tumor models, including *PIK3CA*-mutant breast cancer, *NRAS-* and *BRAF-*mutant melanoma, and neuroblastoma [39, 40].

MONALEESA-1 (NCT01919229) [41] is a phase II study to assess the biological activity of 14 days of neoadjuvant treatment with ribociclib (400 or 600 mg, daily) plus letrozole (2.5 mg, daily), compared with single-agent letrozole (2.5 mg, daily) in postmenopausal patients with newly diagnosed, resectable, HR+, HER2− early breast cancer. The results suggested absence of a drug–drug interaction between ribociclib and letrozole and showed that ribociclib plus letrozole significantly reduced Ki-67 expression in HR+, HER2− breast cancer. MONALEESA-2 (NCT01958021) [42] is a phase III study to evaluate ribociclib as first-line therapy for HR+/HER2− advanced breast cancer. The study group is ribociclib (600 mg/day, 3-week on/1-week off) plus letrozole, compared to single-agent letrozole. Primary endpoint was investigator-assessed progression-free survival. The duration of progression-free survival was significantly longer in the ribociclib group than in the placebo group (hazard ratio, 0.56; 95% CI, 0.43 to 0.72; *p* = 3.29 × 10^−6^ for superiority). Ribociclib received FDA breakthrough therapy designation in combination with letrozole on August 3, 2016, and finally approved on March 13, 2017.

In addition, there are two ongoing phase III trials: MONALEESA-3 (NCT02422615) and MONALEESA-7 (NCT02278120).

MONALEESA-3 is a randomized double-blind, placebo-controlled study of ribociclib in combination with fulvestrant for the treatment of postmenopausal women with hormone receptor-positive (HR+), HER2-negative (HER2−) advanced breast cancer who have received no or only one line of prior endocrine treatment. The phase III MONALEESA-7 study is investigating the combination of ribociclib with goserelin and tamoxifen or nonsteroidal aromatase inhibitor (NSAI) in premenopausal women with HR+/HER2− advanced breast cancer. Patients will be randomly assigned in a 1:1 ratio to two treatment arms: ribociclib or placebo plus goserelin and tamoxifen/NSAI. Furthermore, clinical trials of ribociclib in combination with targeted therapies and/or hormonal therapies are ongoing as well. A phase II trial was conducted based on the preliminary results from a phase I study of ribociclib combined with exemestane and everolimus showing improved efficacy and manageable toxicity [43].

## Abemaciclib

Findings from the phase I study I3Y-MC-JPBA (JPBA) indicate that the abemaciclib single-agent MTD of 200 mg administered orally every 12 h (Q12H) demonstrates an acceptable safety profile. Abemaciclib has demonstrated evidence of clinical activity in women with MBC at doses of both single-agent 150 and 200 mg Q12H, and the range of steady-state exposures is comparable for the two doses. In this study, among 36 patients with HR+ MBC receiving abemaciclib, the median PFS was 8.8 months and there were 12 confirmed partial responses (PRs), for an objective response rate of 33.3%. In the same study, the combination of abemaciclib plus fulvestrant was also evaluated and demonstrated an acceptable safety profile in 19 women with four confirmed PRs observed [44]. Safety and tolerability of abemaciclib in combination with endocrine therapies (including anastrozole and letrozole) are being further evaluated in patients with HR+, human epidermal growth factor receptor 2-negative (HER2−) MBC, in the ongoing phase Ib study I3Y-MC-JPBH (JPBH) [45].

Phase II study (MONARCH-1) has investigated in 132 HR+ metastatic breast cancer patients, further confirming that single-agent abemaciclib at a dose level of 200 mg Q12H is clinically active. In MONARCH-1 study, 19.7% of the overall population has achieved an overall response rate (ORR). The median PFS was 6 months, and the median overall survival (OS) was 17.7 months. 90.2% of the patients had grade 1–3 diarrhea with 70.5% in grade 1 or 2. The decrease in white blood cell had been observed in 90.8% patients, 63.1% of which in grade 1 or 2 [46].

Phase II neoadjuvant study (neoMONARCH) comparing the biological effects of abemaciclib plus anastrozole vs abemaciclib monotherapy vs anastrozole monotherapy in women with early-stage HR+, HER2− BC. Patients (pts) were stratified by progesterone receptor status and tumor size and randomized 1:1:1. The results indicated that abemaciclib, along or in combination with anastrozole, significantly reduced Ki-67 expression compared to anastrozole along after 2 weeks of treatment based on geometric mean change (63.2 vs 92.6 vs 90.6%) and complete cell cycle arrest (14.8 vs 66.1 vs 58.8%). The majority of patients experienced an objective response [47].

Ongoing clinical trials include two randomized, double-blind, placebo-controlled, phase III studies, I3Y-MC-JPBM (MONARCH-3) and I3Y-MC-JPBL (MONARCH-2), to further confirm the safety and efficacy of abemaciclib in combination with current standard endocrine therapies (either NSAIs or fulvestrant) in HR+, HER2− breast cancer. Future directions also include using the CDK4 and CDK6 inhibitors in the adjuvant and neoadjuvant therapy settings. A neoadjuvant trial investigating the combination of abemaciclib and aromatase inhibitor in locally advanced ER-positive, HER2-negative breast cancer (neoMONARCH, NCT02441946) is ongoing [48]. More exciting, abemaciclib distributes efficiently to the brain in nonclinical species, potentially providing a unique opportunity to treat primary brain tumors as well as cancers that have metastasized to the brain (Lilly internal data).

## PD1 and PD-L1 antibodies

In the past several years, immunotherapy has been established as a new standard of care, with remarkable activity and curative potential in patients with a broad range of tumor types. Antibodies to cytotoxic T-lymphocyte antigen 4 (CTLA-4), programmed death-1(PD-1), and programmed death-ligand 1 (PD-L1), all of which increase the immune response against the tumor by blocking immune-regulating proteins that downregulate the immune system, have increased response rates and OS in melanoma, non-small cell lung cancer, renal cell carcinoma, Hodgkin lymphoma, urothelial carcinoma, and squamous cell carcinoma of the head and neck [49–55].

The role of immunotherapy in breast cancer has yet to be defined, but increasing evidence points to triple-negative breast cancer (TNBC) as possibly having unique characteristics that may make them more responsive to checkpoint inhibition. The higher genomic instability and mutational burden of TNBC result in a higher propensity to generate neoantigens, which can be recognized as “nonself” by the adaptive immune system [56].

TNBC have a higher amount of tumor-infiltrating lymphocytes (TILs) [57] and higher PD-L1 protein [58, 59] or messenger RNA (mRNA) [60, 61] expression compared with other breast cancer subtypes. Higher levels of TILs generally are associated with poor-prognostic clinicopathologic features, including ER negativity, higher grade, higher proliferative rate, and lymph node positivity [57, 62–65]. However, despite worse clinical features, higher levels of TILs are associated with improved DFS and OS, independent of systemic therapy [65–67]. An association between greater tumor infiltrative lymphocytes (TILs) and better prognosis in breast cancer has been recognized for some time; newer studies have shown the specific relevance in TNBC, which has been shown to have substantial infiltration with TILs [57, 68–70]. This apparent paradox highlights the role that the immune system may play in a subset of TNBC and suggests that TILs may be a surrogate for an adaptive immune response in these cancers.

PD-L1 expression is significantly associated with the presence of TILs [58–60], correlates with higher histologic grade, greater tumor size, and higher expression of the proliferation marker Ki-67 [71]. Data from The Cancer Genome Atlas (TCGA) have confirmed higher PD-L1 mRNA expression in TNBC vs non-TNBC samples [60]. This and other studies have shown that PD-L1 is not detected in normal breast tissue but is expressed in about half of all breast cancers, including approximately 20 to 30% of TNBCs [72, 73], which suggests that the most common mechanism of regulation of PD-L1 expression in TNBC is regulatory feedback (acquired resistance) to immune engagement. In addition, the loss of PTEN expression in TNBCs is associated with PD-L1 overexpression [60], confirming an association between increased PI3K signaling and the presence of PD-L1 [74]. These findings suggest that, in addition to acquired resistance mechanisms, PD-L1 expression can also be regulated by molecular alterations and oncogenic pathways (intrinsic resistance), linking molecular and immune heterogeneity.

More recently, analysis of gene expression profiles of 587 TNBC samples identified six distinct subtypes, including an immunomodulatory (IM) subtype characterized by high expression of immune-related genes. This subtype is rich of immune-activated and associated signaling components contributed from both the tumor and the infiltrating lymphocytes, and it has been associated with improved relapse-free survival compared with other subtypes [75]. RNA sequencing also showed this subtype to have substantially higher expression of PD-L1, PD-1, and CTLA-4. These and other data provide evidence that IM subset is mostly likely to benefit from checkpoint inhibition.

## Pembrolizumab

Pembrolizumab is a high-affinity, highly selective, humanized monoclonal IgG4 antibody against PD-1 that prevents PD-1 from binding to its ligands, PD-L1 and PD-L2. Pembrolizumab is approved in several countries for the treatment of advanced melanoma [76], non-small cell lung cancer in certain situations [77], and as a second-line treatment for head and neck squamous cell carcinoma [78]. Additionally, clinical studies with pembrolizumab have demonstrated promising efficacy with durable responses and a manageable safety profile in many advanced malignancies, including gastric cancer [79] and urothelial cancer [80].

KEYNOTE-012 (NCT01848834) was a multicenter, non-randomized phase Ib trial of single-agent pembrolizumab given at 10 mg/kg every 2 weeks to patients with advanced PD-L1-positive (expression in stroma or ≥1% of tumor cells by immunohistochemistry) malignancies. Among 111 patients with TNBC whose tumor samples were screened for PD-L1 expression, 58.6% had PD-L1-positive tumors; 32 women were enrolled and assessed for safety and antitumor activity. All patients had metastatic TNBC at study entry, and most were heavily pre-treated, having received therapy in both the early and advanced disease settings. Among the 27 patients who were evaluable for antitumor activity, the ORR was 18.5% (1 CR and 4 PR). The median time to response was 17.9 weeks (range, 7.3 to 32.4 weeks). The most common treatment-related AEs of any grade included arthralgia (18.8%), fatigue (18.8%), myalgia (18.8%), and nausea (15.6%), including 5 (15.6%) patients with grade ≥3 toxicity and 1 treatment-related death [81].

KEYNOTE-028 (NCT02054806) is an ongoing multi-cohort, open-label phase Ib study evaluating the safety and efficacy of pembrolizumab in patients with PD-L1-positive advanced solid tumors. Of the 248 patients with ER+/HER2-negative breast cancer who had evaluable tumor samples screened for PD-L1 expression, 48 (19%) had PD-L1-positive tumors. Of these, 25 patients were enrolled. Patients were heavily pretreated, with 76% having received ≥3 prior lines of therapy for advanced disease, including 48.0% who received ≥5 prior lines. In the 22 patients with at least one scan after baseline, ORR was 14% and CBR was 23%. The safety profile was similar to KEYNOTE-012 with 16.0% grade 3–4 AEs including grade 3 autoimmune hepatitis (4%) [82].

A randomized, phase III study of single-agent pembrolizumab vs single-agent chemotherapy per physician’s choice for metastatic TNBC (KEYNOTE-119/NCT02555657) is ongoing. Estimated 600 metastatic triple-negative breast cancer (mTNBC) patients with central determination of PD-L1 tumor status who has previously received either one or two prior systemic treatments for metastatic setting will receive pembrolizumab 200 mg intravenously every 3 weeks or receive capecitabine, eribulin, gemcitabine, and vinorelbine per physician’s choice. The primary endpoint is PFS and OS. The primary completion date will be this June. Future directions also include using pembrolizumab in the neoadjuvant therapy settings. A neoadjuvant trial investigating pembrolizumab in combination with chemotherapy as neoadjuvant treatment for participants with TNBC is ongoing (KEYNOTE-173/NCT02622074). Estimated 100 previously untreated, locally advanced TNBC will be allocated to received pembrolizumab combined or sequentially with paclitaxel/nab-paclitaxel/doxorubicin/cyclophosphamide/carboplatin. The primary endpoint is DLTs with secondary endpoints being pathologic complete response (pCR) and ORR.

## Atezolizumab

Atezolizumab is an engineered, humanized IgG1 monoclonal antibody that targets PD-L1 and inhibits the interaction between PD-L1 and these two receptors, PD-1 and B7-1. It has been approved in the USA for the treatment of locally advanced or metastatic urothelial carcinoma and metastatic non-small cell lung cancer. Atezolizumab has shown activity in TNBC both as single-agent and combination treatment with chemotherapy in early phase trials and is now being tested in phase III setting.

In a phase I study (NCT01375842, PCD4989g), clinical activity analyses have been performed in 21 patients with PD-L1-selected (IC2/3) TNBC who received atezolizumab treatment at 1200 mg Q3W [73]. Unconfirmed responses were recorded for 5 patients, of whom two experienced a complete response and 3 patients experienced a partial response. As of 2^nd^ September 2014, 4 of these 5 patients were still responding and 1 patient experienced disease progression. The Kaplan–Meier estimated overall 24-week PFS rate was 33% (95% CI 12 to 53%).

Another phase Ib multi-arm study (NCT01633970, GP28328) evaluates the safety and preliminary efficacy of a number of combinations of atezolizumab in patients with locally advanced or metastatic solid tumors. Arm F of the study is testing the combination of atezolizumab and nab-paclitaxel in female patients with metastatic TNBC. Patients received 800 mg of atezolizumab on days 1 and 15 of every 28-day cycle plus nab-paclitaxel (125 mg/m^2^) on days 1, 8, and 15 of every 28-day cycle. Up to two prior cytotoxic regimens for metastatic disease were allowed. By 14 January 2016, safety and preliminary efficacy data were available for 32 patients [83]. Of the efficacy-evaluable patients, 13 received the treatment combination as first-line therapy and 19 had received ≥1 prior cytotoxic regimens for metastatic disease; 88% had previously received taxanes. In the overall efficacy-evaluable population, 12 patients (38%) achieved objective responses. Clinical responses were observed in patients with PD-L1 IC1/2/3 expression tumors as well as in those with PD-L1 IC0 expression. Six of 13 patients (46%) who received atezolizumab plus nab-paclitaxel as first-line therapy achieved responses, comprising one complete response and five partial responses. The combination was well tolerated and consistent with the known risks of nab-paclitaxel and atezolizumab [83]. The most frequent AEs attributed to atezolizumab (≥10%) included fatigue, pyrexia, diarrhea, nausea, alopecia, pruritis, headache, peripheral neuropathy and peripheral sensory neuropathy, and decreased neutrophil count. Based on these results, the combination of atezolizumab and nab-paclitaxel is being evaluated in a phase III study (NCT02425891) of patients with previously untreated mTNBC.

## Nivolumab

As the first PD-1 blocking antibody approved for clinical practice in worldwide, nivolumab has got its indications in unresectable or metastatic melanoma, metastatic non-small cell lung cancer, advanced renal cell carcinoma, classical Hodgkin lymphoma, and recurrent/metastatic squamous cell carcinoma of the head and neck [49, 52–54, 84, 85]. But the clinical data in breast cancer is rare reported. Up to date, no clinical result of significance published on nivolumab-treated breast cancer, but indeed, there are many ongoing trials to assess the safety and efficacy of nivolumab as monotherapy or combined therapy in this disease.

A phase I/II, open-label study of nivolumab as monotherapy or combined with ipilimumab in advanced or metastatic solid tumors with a cohort of TNBC is currently recruiting [86]. The primary purpose is to analyze the safety and efficacy (ORR, PFS). Additionally, evaluation of putative biomarkers such as PD-1 and PD-L1 expression will be performed. The study uses a modified Simon 2-stage design. In stage 1, 36 patients for each tumor type will be assigned 1:1 to treatment with either nivolumab (*N*) or nivolumab + ipilimumab (*N* + *I*) for 4 doses and then nivolumab maintenance until progression or toxicity. Treatment arms will proceed independently into stage 2 if ≥2 patients in a given arm for each tumor type have an OR. In stage 2, an additional 22 patients per tumor type will be assigned to each arm (*N* or *N* + *I*) and receive the stage 1 dosing regimen. The estimated completion date will be December 2018.

In another phase I trial, nivolumab (nivo) is tested combined with nab-paclitaxel (nab-P) in HER2-negative MBC [87]. Patients with MBC will be treated in 2 arms: nab-P 100 mg/m^2^ on days 1, 8, and 15 of each 28-day cycle plus nivo 3 mg/kg on days 1 and 15 starting at cycle 3 or nab-P 260 mg/m2 on day 1 of each 21-day cycle plus nivo 5 mg/kg on day 15 starting at cycle 3. The primary endpoint is the DLTs. Secondary study endpoints include treatment-emergent adverse events (TEAEs), PFS, OS, disease control rate, ORR, and duration of response. Exploratory endpoints include tumor-associated PD-L1 expression, modulation of immune activation in the tumor and peripheral blood in response to nivo treatment, serum nivo levels, and development of antiglobulin antibodies. The estimated completion date will be October 2017.

Other ongoing clinical trials besides those mentioned above are listed in Table 1.

**Table 1:** Clinical trials testing nivolumab in patients with breast cancer

Disease setting	Phase	Clinical trial reference number	Cancer type	Estimated Enrollment	Primary Endpoint	Regimens	Control arm
Metastatic	II	NCT02892734	HER2-	29	PFS	Nivolumab + ipilimumab	None
Metastatic	I	NCT02309177	HER2-	20	MTD	Nivolumab + nab-paclitaxel	None
Metastatic	II	NCT02499367	TNBC	84	PFS	Nivolumab + doxorubicin/cyclophosphamide/cisplatin/radiation	Active Comparator
Metastatic	I	NCT02453620	HER2-	45	Safety	Nivolumab + entinostat + ipilimumab	None
Metastatic	I/II	NCT01928394	TNBC	58	ORR	Nivolumab ± ipilimumab	None
Metastatic	I	NCT02834247	TNBC	36	MTD/ORR	Nivolumab ± TAK659	None

TNBC, triple negative breast cancer; HER2, human epidermal growth factor receptor-2; PFS, progress free survival; MTD, maximum tolerance dose; ORR, objective response rate

## Avelumab

Avelumab (MSB0010718C) is a fully human anti-PD-L1 IgG1 antibody recently approved by FDA for the treatment of metastatic Merkel cell carcinoma [88]. In a cohort of phase Ib JAVELIN study (NCT01772004), 168 patients with locally advanced or metastatic breast cancer refractory to or progressing after standard-of-care therapy received avelumab at 10 mg/kg Q2W [89]. The ORR in the entire cohort was 5.4%, including 5 PRs in TNBC (*n* = 57). Among all patients with PD-L1-expressing immune cells within the tumor, 33.3% (4 of 12) had PRs. In patients with TNBC who had PD-L1+ immune cells within the tumor, 44.4% (4 of 9) had PRs, compared with 2.6% (1 of 39) for TNBC and PD-L1− immune cells. Contrastively, out of the 72 patients with HR+ disease, an objective response was seen in only 2.8%, but 54% were found to have PD-L1 expression. This brings into question the antibodies used for PD-L1 testing as well as potential differences in efficacy between different subsets of breast cancer.

A phase III randomized trial is ongoing to test adjuvant treatment for high-risk TNBC patients with avelumab (A-Brave, NCT02926196). Patients with high-risk primary TNBC (all comers, PD-L1-positive or unselected for PD-L1 status) who have completed treatment with curative intent including surgery of the primary tumor, neo- or adjuvant chemotherapy, and radiotherapy (if indicated) are recruited and randomized to experimental arm (avelumab for 1 year) or no further intervention arm. The primary endpoint is DFS. The primary completion date will be June 2021.

## PARP inhibitors

Healthy cells defend themselves against the deleterious effects of DNA damage through an interrelated series of molecular pathways, the DNA damage response (DDR), that recognize DNA damage, stall the cell cycle, and mediate DNA repair. Poly(ADP-ribose) polymerase (PARP) are nuclear enzymes that catalyze the transfer of ADP ribose from NAD+ to target proteins and facilitate DNA repair [90]. At sites of DNA damage, PARP1 binds damaged DNA at single-strand DNA breaks (SSBs) and other DNA lesions, an event that causes a series of allosteric changes in the structure of PARP1 that activate its catalytic function PARP and activates intracellular signaling pathways that modulate DNA repair and cell survival through poly(ADP)-ribosylation of several nuclear proteins involved in chromatin architecture and DNA metabolism [91–93]. PARP inhibition results in double-strand breaks in replicating cells [94]. In cells with wild-type BRCA1/2, double-strand breaks are repaired via homologous recombination, but in BRCA1/2-deficient cells with homologous recombination deficiency (HRD), DNA strand breaks rely on PARP1 functionality for repair [94, 95]. Therefore, inhibition of PARP1 by RNA interference or with chemical inhibitors leads to severe, highly selective toxicity in BRCA1/2-deficient cells [96], the so-called synthetic lethality [97]. In breast cancer, the presence of germline mutations in BRCA1/2 is characterized by features of basal-like sporadic breast tumors, including a greater likelihood of being high-grade, ER/PgR-negative, HER2-negative, and a high frequency of TP53 mutations [98]. The presence of germline mutations in BRCA1/2 increases the lifetime risk of breast cancer to 60–70% [99] and occurs in about 10% of patients with TNBC [18, 100, 101].

Up to date, three typical PARP inhibitors—olaparib [102, 103], rucaparib [104, 105], and niraparib [106], have all received their FDA approval for advanced ovarian cancer and/or primary peritoneal cancer with or without germline and/or somatic mutations in BRCA1/2. In the setting of breast cancer, a proof of concept study was conducted to assess the efficacy, safety, and tolerability of olaparib alone in women with BRCA1 or BRCA2 mutation advanced breast cancer. Patients had been given a median of three previous chemotherapy regimens. Overall responses ranged from 22% (6 out of 27, 100 mg twice per day) to 41% (11 out of 27, 400 mg twice per day) with tolerable toxicity [107]. However, a phase II study evaluating olaparib 400 mg twice a day as a single agent for patients with advanced breast cancer (*n* = 26, 81% TNBC) did not report any confirmed responses in BRCA1/2 mutation neither positive (*n* = 10) nor negative (*n* = 16) subjects, even though the target lesions were reduced in size by >30% in 5 out of 10 (50%) patients with BRCA1 or BRCA2 mutations, but were not confirmed objective responders because of absence of confirmation at the next visit (three patients) or progression of nontarget or new lesions at the same visit (two patients) [108]. A phase I/Ib study tested the effects, safety, and activity of the combination of olaparib and carboplatin. Olaparib tablets were introduced in a 3 + 3 dose escalation with carboplatin q21 days, up to 8 cycles, followed by olaparib 300 mg bid maintenance. Fourteen patients with breast cancer (11 TNBC, 7 germline BRCA mutation carriers) were enrolled. One patient with BRCA1 mutation TNBC achieved CR and another 6 achieved PR [109].

Several randomized phase III trials investigating the use of olaparib in the metastatic (NCT02000622) and neoadjuvant (NCT02032823) setting are ongoing. Germline mutation in BRCA1 or BRCA2 is essential as an inclusion criterion in these trials. The primary endpoints are PFS and invasive disease-free survival (IDFS), respectively. Another PARP inhibitor, talazoparib, is studied in neoadjuvant setting. Thirteen early-stage breast cancer patients with germline mutations in either BRCA1 or BRCA2 were treated for 2 months with talazoparib. All patients displayed a reduction in tumor volume from 30 to 98% after 2 months [110]. This study is now being expanded to assess the effects of 4 to 6 months of neoadjuvant talazoparib therapy. Similar neoadjuvant studies assessing rucaparib in breast cancer are also under way.

## Fulvestrant

Fulvestrant is a new type of endocrine treatment—an ER antagonist with a novel mode of action. Fulvestrant is a 7a-alkylsulphinyl analog of 17b-oestradiol, which is distinctly different in chemical structure from the nonsteroidal structures of tamoxifen, raloxifene, and other SERMs [111].

Fulvestrant competitively inhibits binding of estradiol to the ER, with a binding affinity of 89% of estradiol, while tamoxifen affinity is only 2.5% of that of estradiol [112, 113]. Fulvestrant–ER binding impairs receptor dimerization, and energy-dependent nucleo-cytoplasmic shuttling, thereby blocking nuclear localization of the receptor [114, 115]. Additionally, any fulvestrant–ER complex that enters the nucleus is transcriptionally inactive and has no demonstrable agonist activity because both AF1 and AF2 are disabled. Finally, the fulvestrant–ER complex is unstable, resulting in accelerated degradation of the ER protein, compared with estradiol- or tamoxifen-bound ER [116], leading to complete inhibition of estrogen signaling [117–119]. Therefore, it is also called as selective estrogen receptor downregulator.

The phase I clinical trials in postmenopausal women with primary breast cancer have shown that fulvestrant significantly downregulates ER expression in ER-positive tumors in a dose-dependent manner. There was also a significant decrease in progesterone receptor (PR) expression (a marker of estrogen action) consistent with the preclinical data demonstrating that fulvestrant lacks intrinsic estrogen agonist activity. These changes in ER and PR expression were accompanied by reductions in expression of Ki-67, a marker of tumor cell proliferation [120]. Neoadjuvant NEWEST trial showed that in 211 early breast cancer patients, the 500 mg regimen of fulvestrant resulted in a significantly (*p* < 0.0003) greater reduction in ER expression compared with the 250-mg dose at week 4 (22 vs 15%) [121]. At week 16, ER expression was reduced by 34 and 25%, respectively [121].

Four phase III clinical trials (studies 9238IL/0020 and 9238IL/0021) [122, 123], EFECT [124] and SoFEA [125] showed that fulvestrant 250 mg is as effective as the conventionally used drugs. Fulvestrant 250 mg combined with AI gave contradictory results compared with AI only in two phase III trials [126, 127].

Increase of dose of fulvestrant improves efficacy. CONFIRM trial [128] was completed in 736 postmenopausal women with advanced breast cancer who had disease recurrence on or after adjuvant endocrine therapy or progression following endocrine therapy for advanced disease. Fulvestrant 500 mg significantly improves median PFS, which translating into a 4.1-month increase in median OS and a 19% reduction in the risk of death. First-line randomized phase II trial in 205 postmenopausal women with fulvestrant 500 mg treatment had a median PFS and OS of 23.4 and 54.1 m, respectively [129]. The confirmative phase III FALCON (NCT01602380) trial showed that patients treated with fulvestrant had a statistically significant 21% improvement in progression-free survival compared to those treated with anastrozole (16.6 vs 13.8 months, *p* = 0.048) [130]. Fulvestrant ongoing clinical studies are listed in Table 2. Other SERDs, such as AZD9291, AZD9496, and GDC810 are under clinical investigations.

**Table 2:** Fulvestrant ongoing clinical studies

CT.gov No.	Name	Investigational Agent	Trial
NCT02646735	FRIEND	Fulvestrant	A Randomized, Open label, Parallel-group, Multi-Centre, Pilot study to compare the Efficacy and Tolerability of Fulvestrant 500mg with Exemestane as First line endocrine therapy for Postmenopausal Hormone Receptor Positive HER2 negative Advanced Breast Cancer patients relapse after adjuvant Non-steroidal Aromatase Inhibitors
		Exemestane	
NCT02072512	PROOF	Goserelin	A Phase III, Randomized, Open label, Parallel-group, Multi-Centre study to compare the Efficacy of Goserelin combined with Fulvestrant 500 mg and Anastrozole 1mg as First line endocrine therapy for Pre- or Peri-menopausal patients having Hormone Receptor Positive Advanced Breast Cancer After or During Adjuvant Endocrine therapy
		Fulvestrant	
		Anastrozole	
NCT02107703	MONARCH 2	Fulvestrant	A Randomized, Double-Blind, Placebo-Controlled, Phase 3 Study of Fulvestrant With or Without Abemaciclib, a CDK4/6 Inhibitor, for Women With Hormone Receptor Positive, HER2 Negative Locally Advanced or Metastatic Breast Cancer
		Abemaciclib	
NCT02422615	MONALEESA 3	Ribociclib	A Randomized Double-blind, Placebo-controlled Study of Ribociclib in Combination With Fulvestrant for the Treatment of Men and Postmenopausal Women With Hormone Receptor Positive, HER2-negative, Advanced Breast Cancer Who Have Received no or Only One Line of Prior Endocrine Treatment
		Fulvestrant	
NCT02690480	FLIPPER	Fulvestrant	A Randomized, Double-blind, Parallel-group, Multicentre, Phase II Study to Compare the Efficacy and Tolerability of Fulvestrant 500mg With Placebo and Fulvestrant 500mg in Combination With Palbociclib as First Line Treatment for Postmenopausal Women With Hormone Receptor-positive Metastatic Breast Cancer, Who Have Completed at Least 5 Years of Adjuvant Endocrine Therapy and Remained Disease Free for More Than 12 Months Following Its Completion or Have “de Novo” Metastatic Disease
		Palbociclib	
NCT02028507	PEARL	Palbociclib	Phase III Study of Palbociclib in Combination With Endocrine Therapy (Exemestane or Fulvestrant) Versus Chemotherapy (Capecitabine) in Hormonal Receptor (HR) Positive/HER2 Negative Metastatic Breast Cancer (MBC) Patients With Resistance to Non-steroidal Aromatase Inhibitors
		Exemestane	
		Fulvestrant	
		Capecitabine	
NCT02491983	PARSIFAL	Palbociclib	A Randomized, Multicenter, Open-label, Phase II Trial to Evaluate the Efficacy and Safety of Palbociclib in Combination With Fulvestrant or Letrozole in Patients With HER2 Negative, ER+ Metastatic Breast Cancer
		Fulvestrant	
		Letrozole	
NCT02536742	PYTHIA	Palbociclib	A Phase II Study of Palbociclib Plus Fulvestrant Versus Placebo Plus Fulvestrant for Pretreated Patients With ER+/HER2- Metastatic Breast Cancer
		Fulvestrant	
NCT01633060	BELLE 3	BKM120	A Phase III Randomized, Double Blind Placebo Controlled Study of BKM120 With Fulvestrant, in Postmenopausal Women With Hormone Receptor-positive HER2-negative Locally Advanced or Metastatic Breast Cancer Which Progressed on or After Aromatase Inhibitor Treatment
		Fulvestrant	
NCT02340221	SANDPIPER	Taselisib	A Phase III, Double-Blind, Placebo Controlled, Randomized Study of Taselisib plus Fulvestrant vs Placebo plus Fulvestrant in Postmenopausal women with Estrogen Receptor-Positive and HER2-Negative Locally Advanced or Metastatic Breast Cancer who have Disease Recurrence or Progression during or after Aromatase Inhibitor Therapy
		Fulvestrant	
NCT02216786	MANTA	Fulvestrant	A Randomized Phase II Study of Fulvestrant in Combination With the Dual mTOR Inhibitor AZD2014 or Everolimus or Fulvestrant Alone in Estrogen Receptor-positive Advanced or Metastatic Breast Cancer ()
		AZD2014	
		Everolimus	

## T-DM1 and other ADCs

Approximately 18–20% of invasive breast cancers are HER2-positive subtype with poor prognosis in the absence of anti-HER2 treatment. Trastuzumab emtansine (T-DM1) is a complex compound produced by the conjugation of trastuzumab, a stable thioether linker, and the potent cytotoxic drug maytansine derivate (DM1). It is the first antibody-drug conjugate (ADC) developed specifically for the treatment of HER2-positive breast cancer [131, 132]. The binding of T-DM1 to HER2-positive cells allows internalization of this complex by endocytosis, subsequent intralysosomal proteolytic degradation, and then release of potent DM1, a derivative of the antimitotic drug maytansine [133, 134].

The maximum tolerated dose (MTD) determined by the phase I and II clinical trials is 3.6 mg/kg every 3 weeks with bone marrow suppression and liver toxicity being dose-limiting toxicity, the clearance of the drug is 12.9 ml/day/kg (±3.4 ml/day/kg) and its half-life is 3.5 days [135, 136].

The first-line MARIANNE trial (NCT01120184) is a large three-arm phase III study which randomized patients with previously untreated HER2-positive MBC to receive T-DM1 plus pertuzumab,T-DM1 plus placebo, and combination of trastuzumab plus a taxane (paclitaxel or docetaxel). T-DM1 is concluded to be non-inferior to trastuzumab + taxanes but with a better toxicity profile [137].

The two pivotal trials, EMILIA and TH3RESA trials conducted in second line and third- and later lines, respectively, demonstrated that T-DM1 is better than lapatinib/capecitabine and treatment of physician’s choice (TPC), respectively, in terms of ORR and PFS as well as OS. These two phase III trials suggest that T-DM1 is standard of choice in second- and later-line management of HER2-positive MBC.

The ongoing studies, including phase Ib and II studies, STELA, BP22572, TDM4529g/B025430, and TDM4874g/BO22857 in adjuvant/neoadjuvant setting and TEAL in neoadjuvant setting, and phase III studies, MO28231 in metastasis setting, KAITLIN (B028407) and KATHERINE (BO27938) in adjuvant setting, and KRISTINE (BO28408/TRIO021) in neoadjuvant setting, will help to elucidate if T-DM1 could have a role in first-line treatment of metastatic breast cancer as well as in the adjuvant and neoadjuvant setting.

ADCs are biological drugs containing a monoclonal antibody linked by a covalent bond to a cytotoxic drug via a synthetic coupler. The ADC is designed such that when it reaches the target cell, it releases the cytotoxic agent inside them, thus sparing non-tumor cells from damage. In preclinical experiment, the new triple conjugate, T-DM1 with another antibody, such as pertuzumab or atezolizumab, was successful and works in cell lines as well as animal models.

## Innovative chemotherapies

### Nab-paclitaxel

Taxanes are widely used as antitumor agents. Albumin-bound paclitaxel (nab-paclitaxel; Abraxane) is a second generation of taxanes, that has been developed to improve the therapeutic index of paclitaxel, also reducing the toxicities associated with Taxol and the CrEL and ethanol vehicle. Nab-paclitaxel is a good candidate since it can be given without steroid or antihistamine premedication. Due to its safety, nab-paclitaxel can be delivered at higher doses, in a shorter infusion time, thus enabling a higher drug Cmax and plasma area under the curve (AUC). Upon intravenous infusion, nab-paclitaxel dissociates into its albumin and paclitaxel on small particles of 8–30 nm and then distributes rapidly to extravascular compartment and selectively delivers larger amounts of nab-paclitaxel to tumors by exploiting endogenous albumin transport pathways [138, 139].

Nab-paclitaxel was approved for metastatic breast cancer by FDA in 2005. Since then, it has been studied in a variety of breast cancer patient populations and with different doses and schedules. The GeparSepto (GBG 69) trial assessed weekly nab-paclitaxel on improving pathological complete response rate compared with weekly solvent-based paclitaxel, both followed by epirubicin plus cyclophosphamide as neoadjuvant treatment. Results showed that 12 continuous weekly doses of nab-paclitaxel 125 mg/m^2^ for neoadjuvant therapy is both well tolerated and associated with significant superior pCR rates (38%) vs weekly paclitaxel 80 mg/m^2^ (29%) [140]. This result is consistent with that of another phase III ETNA study [141].

In metastatic setting, the phase II tnAcity study results were presented in 2016 SABCS meeting. One hundred ninety-one women with mTNBC were randomized to receive one of three weekly regimens: nab-paclitaxel + carboplatin (nab-P/C), nab-paclitaxel + gemcitabine (nab-P/G), or gemcitabine + carboplatin (G/C) as first-line treatment. The trial found that an investigational weekly combination regimen of nab-P/C had significantly longer PFS (7.4 months) compared to weekly regimens of either nab-P/G (5.4 months; *p* = 0.02) or G/C (6.0 months; *p* = 0.03) [142]. The approval in MBC was based on a randomized phase III trial of nab-paclitaxel 260 mg/m^2^ vs paclitaxel 175 mg/m^2^ every 3 weeks. Nab-paclitaxel demonstrated a significantly higher overall response rate (ORR 33 vs 19%; *p* = 0.001) and longer time to tumor progression (23 vs 17 weeks; HR 0.75; *p* = 0.006) vs paclitaxel in the intention-to-treat (ITT) population [143].

A systematic review discussed recent studies and ongoing trials of nab-paclitaxel in breast cancer and provides perspectives on the future role of nab-paclitaxel in breast cancer. Sixty-three studies of nab-paclitaxel in breast cancer published between 2013 and 2015 were analyzed, including 23 in early stage and 30 in metastatic setting. Among phase II and III studies of neoadjuvant nab-paclitaxel (majority administered weekly) that did not select for specific disease subtype, the pCR rate ranged from 22 to 40%. And for HER2-negative breast cancer or TNBC, the overall pCR rate ranged from 5.7 to 53% with the highest pCR rate achieved in TNBC treated by nab-paclitaxel + carboplatin. Four studies of nab-paclitaxel in MBC of unselected subtype reported median OS ranging from 10.8 months with nab-paclitaxel 260 mg/m^2^ q3w to 26.9 months with nab-paclitaxel 125 mg/m^2^ qw 3/4 combined with cisplatin. Response rate by subgroup demonstrated a higher response in TNBC [144].

Nab-paclitaxel is continuously being investigated in different stages and settings of aggressive breast cancer listed in Table 3. Immune checkpoint inhibitors and their optimal combination partners are hot topics [145].

**Table 3:** Nab-paclitaxel’s ongoing trials phase III and important phase II trials as listed below

CT.gov No.	Phase	Investigational Agent	Trial	Setting	n	Completion Date
NCT02620280	III	Atezolizumab	Neo-Adjuvant Study With the PDL1-directed Antibody in Triple Negative Locally Advanced Breast Cancer Undergoing Treatment With Nab-paclitaxel and Carboplatin (NeoTRIPaPDL1)	Neoadjuvant TNBC	272	2022
NCT02425891	III	Atezolizumab	A study of atezolizumab in combination with *nab*-paclitaxel compared with placebo with *nab*-paclitaxel for participants with previously untreated metastatic triple negative breast cancer (IMpassion130)	mTNBC	900	2020
NCT02819518	III	Pembrolizumab	A Randomized, Double-Blind, Phase III Study of Pembrolizumab (MK-3475) Plus Chemotherapy vs Placebo Plus Chemotherapy for Previously Untreated Locally Recurrent Inoperable or Metastatic Triple Negative Breast Cancer - (KEYNOTE-355)	Locally recurrent or metastatic BC	858	2019
NCT01690702	III	Epirubicin, Cyclophosphamide Docetaxel	Adjuvant Phase III Trial to Compare Intense Dose-dense Adjuvant Treatment With EnPC to Dose Dense, Tailored Therapy With dtEC-dtD for Patients With High-risk Early Breast Cancer (GAIN-2)	Adjuvant high risk BC	2886	2020
CBCSG018	II	Gemcitabine	A randomized phase 2 trial of weekly nab-paclitaxel plus cisplatin versus gemcitabine plus cisplatin as first-line treatment for patients with metastatic triple-negative breast cancer	mTNBC	254	2018
		Cisplatin				
NCT02685059	II	Durvalumab	A Randomized Phase II Study to Investigate the Addition of PD-L1 Antibody MEDI4736 to a Taxane-anthracycline Containing Chemotherapy in Triple Negative Breast Cancer (GeparNuevo)	Neoadjuvant TNBC	174	2018
NCT02783222	II	nab-paclitaxel	A Randomized Phase II Study to Evaluate the EFficacy and Impact on Function of Two Different Doses of Nab-paclitaxEl in Elderly Patients With advanCed breasT Cancer (EFFECT)	≥65y Locally recurrent or metastatic BC	156	2017

## Eribulin

Eribulin mesylate (E7389) is a structurally simplified synthetic analog of halichondrin B, which was first isolated more than 20 years ago from two unrelated species of sponge, *Halichondria okadai* Kadota, and *Aninella* sp. [146, 147]. It is a nontaxane inhibitor of microtubule dynamics and the only cytotoxic agent in the last decade to improve overall survival in heavily pretreated patients with MBC. Eribulin inhibits microtubule polymerization (or growth), through an eribulin-specific binding site on β-tubulin, without any effect on microtubule depolymerization (or shortening) unlike conventional anti-tubulin agents, like taxanes, epothilones, and vinca alkaloids [148]. It may have additional antitumor mechanism through effects on epithelial-to-mesenchymal transition [149] and tumor vasculature remodeling [150, 151].

The first reported phase III study was the EMBRACE (the Eisai Metastatic Breast Cancer Study Assessing Physician’s Choice Versus E7389) [152], the pivotal phase III trial that led to the regulatory approval of eribulin for the treatment of MBC. In this study, 762 women were randomly assigned (2:1) to either eribulin (*n* = 508) or treatment of physician’s choice (TPC; *n* = 254). OS and PFS were the co-primary endpoints. Median overall survival was significantly improved in women assigned to eribulin compared with TPC (13.1 vs 10.6 months, *p* = 0.041). In the early-line MBC setting, eribulin did not improve PFS or OS than capecitabine. Subgroup analysis of the two trials showed that TNBC patients might benefit more from it [153, 154]. A recent trial comparing eribulin head to head with vinorelbine conducted in Chinese population showed that it improved progression-free survival.

Eribulin is currently being studied in several clinical trials. A phase III study comparing eribulin with paclitaxel in the first-line and second-line treatment of HER2-negative MBC is currently recruiting patients in the USA. A phase II study of eribulin in combination with trastuzumab and pertuzumab is currently recruiting (NCT01912963). PD-L1 is expressed in approximately 60% of TNBC tumors, suggesting that PD-L1 may be a therapeutic target for this disease [81]. The combination of pembrolizumab and eribulin demonstrated a 33.3% ORR for patients with metastatic triple-negative breast cancer (TNBC) who received 0 to 2 prior lines of therapy [155]; a further confirmative phase III trial is warranted.

Future research is needed to optimize the role of eribulin in the treatment of MBC, in terms of both patient selection and its position in the therapeutic sequence. Eribulin should also be further tested as first-line treatment in advanced breast cancer, in the adjuvant and neoadjuvant setting alone and in combination with a variety of agents, particularly biologics.

## Utidelone

Refractory to anthracycline and taxane remains a main cause of disease progression for metastatic breast cancer. Epothilones are a class of naturally existing microtubule inhibitors produced by the myxobacterium *Sorangium cellulosum*. The molecular structure and mechanism of action of epothilones differ from those of taxanes. Thus, patients with tumors resistant to taxanes remain sensitive to epothilones [156]. Utidelone is a genetically engineered epothilone analog which attempts to achieve better efficacy, more favorable safety profile, and lower cost than ixabepilone, a semisynthetic epothilone analog which is the only drug in this class that has been approved by the US FDA.

A series of trials have shown promise efficacy for utidelone as a potential treatment for heavily pretreated drug-resistant, advanced breast cancer.

The pivot study is a phase III open-label, superiority, randomized study to enroll patients with metastatic breast cancer refractory to anthracycline and taxane chemotherapy regimens. Four hundred five patients were randomized by 2:1 to treatment with utidelone (30 mg/m^2^ once per day on days 1–5) plus capecitabine (1000 mg/m^2^ twice per day on days 1–14) or capecitabine alone (1250 mg/m^2^ twice per day on days 1–14). The primary endpoints centrally assessed by a masked independent radiology review committee showed improved ORR in the utidelone plus capecitabine group than in the capecitabine alone group (40.4 vs 21.5%; *p* = 0.0002). Median PFS was 8.44 months compared with 4.27 months, respectively (HR 0.46; *p* < 0.0001). The analysis of OS is immature, and analysis with available data by the cutoff date showed longer OS in the utidelone plus capecitabine group compared with the capecitabine alone group (16.13 vs 12.78 months; HR 0.63 *p* = 0.0059). No significant between-group differences were noted for safety outcomes, except for peripheral neuropathy which was significantly higher with utidelone plus capecitabine compared with capecitabine alone (grade 3: 22 vs <1%). Notably, utidelone caused only very mild myelosuppression (leucopenia 48 vs 47% in all grade) and no liver toxicities [157]. Further research is needed to optimize the formulation of utidelone for more convenient administration and to reduce the incidence of peripheral neuropathy. Future development for the role of utidelone in earlier settings of breast cancer, combination studies with other biological immunotherapies, and targeted agents are warranted.

## Other potential agents/therapies

Chimeric antigen receptor (CAR) is a modular fusion protein comprising extracellular target-binding domain usually derived from the single-chain variable fragment (scFv) of antibody, spacer domain, transmembrane domain, and intracellular signaling domain [158]. CAR-engineered T cells (CAR-T cells) have yielded unprecedented efficacy in B cell malignancies, most remarkably in anti-CD19 CAR-T cells for B cell acute lymphoblastic leukemia (B-ALL) with up to a 90% complete remission rate [159, 160]. However, this success has encountered significant hurdles in translation to solid tumors.

Folate receptor-alpha (FRα) is a glycosyl-phosphatidyl inositol (GPI)-anchored protein that is overexpressed at both the protein and mRNA levels in TNBC [161], where it serves a biological role in TNBC cell growth and folate uptake. Strong FRα immunohistochemical staining is highly associated with poor outcome in breast cancer patients [162]. FRα also expressed at low levels on the apical surface of a subset of polarized epithelial cells including the parotid, kidney, lung, thyroid, and breast. Specific overexpression of FRα in certain malignancies, including TNBC, with low coordinate expression in normal tissue, makes FRα an attractive target. The transfer of T cells genetically redirected with a CAR specific for FRα is an attractive technology that is actively being investigated. The CAR approach combines the antigen specificity of an antibody with the ability of T cells to mediate the killing of tumor cells in a single fusion molecule. CAR-modified T cells actively and specifically target their specified antigen and have the capacity to persist as memory cells in vivo [163, 164]. Song et al. demonstrated that FRα-specific CAR-T cells have the capacity to inhibit human TNBC growth in vivo: infused FRα-specific CAR-T cells mediated significant, albeit modest, reduction in tumor progression compared to the control mice treated with untransduced T cells (*p* = 0.01) or with anti-CD19 CAR-T cells (*p* = 0.035), as measured by caliper-based tumor size. The same dose of FRα CAR-T cells mediated more effective tumor regression in mice with MDA-231. FRα tumors, despite larger initial tumor burden, suggest that the regression of TNBC mediated by CAR-T cells is dependent on a sufficient level of surface tumor antigen expression [165]. Future studies will be required to determine the minimal and maximal threshold of FRα expression for activation and effective lysis by FRα CAR-T cells upon stimulation with the TNBC cell lines or autologous tumor cells. Such results might aid in determining which patients may best benefit from FRα CAR-T cell therapy.

MicroRNAs (miRNAs) are small non-coding RNAs and negatively regulate protein-coding gene expressions by promotion of mRNA degradation or inhibition of translation. Overexpressions of oncogenic miRNAs that inhibit tumor suppressor genes are associated with cancer development. On the other hand, reduction or loss of expression of tumor-suppressive miRNAs induce upregulated expression of their target oncogenes [166]. In breast cancer, some miRNAs have been shown to upregulate the functions of oncogenes while others stimulate tumor suppressors. And the various breast cancer subtypes exhibit different molecular miRNA signatures. For instance, miR-342 was expressed most strongly in the ER-positive/HER2-positive tumors [167]. miR-342 influences the ER expression level and the response to tamoxifen [168, 169]. MiR-10b, miR-26a, and miR-153 have been suggested to be potential biomarkers of TNBC [170]. Lehmann et al. revealed that TNBC can be classified into at least six distinct molecular subtypes with differing biological characteristics based on mRNA profiling, including two basal-like types (BL1 and BL2), an immunomodulatory type (IM), a mesenchymal type (M), a mesenchymal stem-like type (MSL), and a luminal androgen receptor type (LAR) [75]. miRNAs also have important roles in endocrine resistance, and some studies have attempted to identify miRNAs that contribute to the clinical benefits of hormonal therapies. The miR-221/222 cluster is associated with tamoxifen resistance in breast cancer cells [171, 172]. Masri et al. suggested that miR-128a modulates the transforming growth factor-β signaling and survival of letrozole-resistant cell lines [173]. Jung et al. suggested that the plasma miR-210 level is useful for predicting and/or monitoring the therapeutic response to treatments involving trastuzumab, and the upregulation of miR-21 expression has been reported to be associated with trastuzumab resistance in HER2-positive breast cancer [174]. Furthermore, Moskwa et al. suggested that miR-182 downregulates BRCA1 expression and found that the manipulation of miR-182 expression in breast cell lines affected their sensitivity to PARP1 inhibitors [175]. miRNA might also contribute to the immune system in breast cancer. Iliopoulos et al. demonstrated that miR-21 expression was upregulated by ovalbumin stimulation in T cells and also that the inhibition of PD-1 increased miR-21 expression [176]. Modulating miRNA expression appears to be a promising strategy for cancer therapy. Specific knockdown of miR-20b in a breast cancer nude mice model has shown to suppress tumor growth in vivo. Systemic delivery of poly-lacticco-glycolic acid-based miR-21 and miR-10b antagonists in a breast cancer model caused dramatic effects on tumor regression [177]. Further biological research into the ability of novel agents to regulate miRNA expression is warranted, and miRNA is expected to become a therapeutic target of treatments for breast cancer.

## Conclusions

Undoubtedly, HER2 and CDK4/6 are the two most important targets for breast cancer; biologicals targeted against the two targets not only increase objective response rates but also prolong PFS. Overall survival improvement is documented for anti-HER2 treatments and has not been determined with CDK4/6 inhibitors. Chemotherapy and endocrine therapy are still the basic treatments, although optimization of dosage remains an unmet need. For triple-negative breast cancer where anti-HER2 and endocrine treatment fail, immunotherapy based on check point inhibitors is promising, especially when combined with chemotherapy.

## References

[1] Leung BS, Potter AH (1987). Mode of estrogen action on cell proliferation in CAMA-1 cells: II. Sensitivity of G1 phase population. J Cell Biochem.

[2] Sutherland RL, Hall RE, Taylor IW (1983). Cell proliferation kinetics of MCF-7 human mammary carcinoma cells in culture and effects of tamoxifen on exponentially growing and plateau-phase cells. Cancer Res.

[3] Sherr CJ (1996). Cancer cell cycles. Science.

[4] Roussel MF, Theodoras AM, Pagano M, Sherr CJ (1995). Rescue of defective mitogenic signaling by D-type cyclins. Proc Natl Acad Sci U S A.

[5] Jiang H, Chou HS, Zhu L (1998). Requirement of cyclin E-Cdk2 inhibition in p16(INK4a)-mediated growth suppression. Mol Cell Biol.

[6] Weinberg RA (1995). The retinoblastoma protein and cell cycle control. Cell.

[7] Shapiro GI (2006). Cyclin-dependent kinase pathways as targets for cancer treatment. J Clin Oncol.

[8] Fry DW, Harvey PJ, Keller PR, Elliott WL, Meade M, Trachet E, Albassam M, Zheng X, Leopold WR, Pryer NK, Toogood PL (2004). Specific inhibition of cyclin-dependent kinase 4/6 by PD 0332991 and associated antitumor activity in human tumor xenografts. Mol Cancer Ther.

[9] Altenburg JD, Farag SS (2015). The potential role of PD0332991 (palbociclib) in the treatment of multiple myeloma. Expert Opin Investig Drugs.

[10] Finn RS, Dering J, Conklin D, Kalous O, Cohen DJ, Desai AJ, Ginther C, Atefi M, Chen I, Fowst C (2009). PD 0332991, a selective cyclin D kinase 4/6 inhibitor, preferentially inhibits proliferation of luminal estrogen receptor-positive human breast cancer cell lines in vitro. Breast Cancer Res.

[11] Wardell SE, Ellis MJ, Alley HM, Eisele K, VanArsdale T, Dann SG, Arndt KT, Primeau T, Griffin E, Shao J (2015). Efficacy of SERD/SERM hybrid-CDK4/6 inhibitor combinations in models of endocrine therapy-resistant breast cancer. Clin Cancer Res.

[12] Hosford SR, Miller TW (2014). Clinical potential of novel therapeutic targets in breast cancer: CDK4/6, Src, JAK/STAT, PARP, HDAC, and PI3K/AKT/mTOR pathways. Pharmgenomics Pers Med.

[13] Qin G, Xu F, Qin T, Zheng Q, Shi D, Xia W, Tian Y, Tang Y, Wang J, Xiao X (2015). Palbociclib inhibits epithelial-mesenchymal transition and metastasis in breast cancer via c-Jun/COX-2 signaling pathway. Oncotarget.

[14] Bocca C, Ievolella M, Autelli R, Motta M, Mosso L, Torchio B, Bozzo F, Cannito S, Paternostro C, Colombatto S (2014). Expression of Cox-2 in human breast cancer cells as a critical determinant of epithelial-to-mesenchymal transition and invasiveness. Expert Opin Ther Targets.

[15] Anders L, Ke N, Hydbring P, Choi YJ, Widlund HR, Chick JM, Zhai H, Vidal M, Gygi SP, Braun P (2011). A systematic screen for CDK4/6 substrates links FOXM1 phosphorylation to senescence suppression in cancer cells. Cancer Cell.

[16] Morikawa A, Henry NL (2015). Palbociclib for the treatment of estrogen receptor-positive, HER2-negative metastatic breast cancer. Clin Cancer Res.

[17] Schroder LB, McDonald KL (2015). CDK4/6 inhibitor PD0332991 in glioblastoma treatment: does it have a future?. Front Oncol.

[18] Cancer Genome Atlas Network (2012). Comprehensive molecular portraits of human breast tumours. Nature.

[19] Geradts J, Wilson PA (1996). High frequency of aberrant p16(INK4A) expression in human breast cancer. Am J Pathol.

[20] Tamura K (2015). Development of cell-cycle checkpoint therapy for solid tumors. Jpn J Clin Oncol.

[21] Shah PD, Dickler MN (2014). Endocrine therapy for advanced breast cancer. Clin Adv Hematol Oncol.

[22] Johnston SR (2010). New strategies in estrogen receptor-positive breast cancer. Clin Cancer Res.

[23] Lukas J, Bartkova J, Bartek J (1996). Convergence of mitogenic signalling cascades from diverse classes of receptors at the cyclin D-cyclin-dependent kinase-pRb-controlled G1 checkpoint. Mol Cell Biol.

[24] Prall OW, Rogan EM, Sutherland RL (1998). Estrogen regulation of cell cycle progression in breast cancer cells. J Steroid Biochem Mol Biol.

[25] Dean JL, Thangavel C, McClendon AK, Reed CA, Knudsen ES (2010). Therapeutic CDK4/6 inhibition in breast cancer: key mechanisms of response and failure. Oncogene.

[26] Nagaraj G, Ma C (2015). Revisiting the estrogen receptor pathway and its role in endocrine therapy for postmenopausal women with estrogen receptor-positive metastatic breast cancer. Breast Cancer Res Treat.

[27] Mayer EL (2015). Targeting breast cancer with CDK inhibitors. Curr Oncol Rep.

[28] Thangavel C, Dean JL, Ertel A, Knudsen KE, Aldaz CM, Witkiewicz AK, Clarke R, Knudsen ES (2011). Therapeutically activating RB: reestablishing cell cycle control in endocrine therapy-resistant breast cancer. Endocr Relat Cancer.

[29] Schwartz GK, LoRusso PM, Dickson MA, Randolph SS, Shaik MN, Wilner KD, Courtney R, O'Dwyer PJ (2011). Phase I study of PD 0332991, a cyclin-dependent kinase inhibitor, administered in 3-week cycles (schedule 2/1). Br J Cancer.

[30] Flaherty KT, Lorusso PM, Demichele A, Abramson VG, Courtney R, Randolph SS, Shaik MN, Wilner KD, O'Dwyer PJ, Schwartz GK (2012). Phase I, dose-escalation trial of the oral cyclin-dependent kinase 4/6 inhibitor PD 0332991, administered using a 21-day schedule in patients with advanced cancer. Clin Cancer Res.

[31] Tamura K, Mukai H, Naito Y, Yonemori K, Kodaira M, Tanabe Y, Yamamoto N, Osera S, Sasaki M, Mori Y (2016). Phase I study of palbociclib, a cyclin-dependent kinase 4/6 inhibitor, in Japanese patients. Cancer Sci.

[32] DeMichele A, Clark AS, Tan KS, Heitjan DF, Gramlich K, Gallagher M, Lal P, Feldman M, Zhang P, Colameco C (2015). CDK 4/6 inhibitor palbociclib (PD0332991) in Rb + advanced breast cancer: phase II activity, safety, and predictive biomarker assessment. Clin Cancer Res.

[33] Finn R, Hurvitz S, Allison M, Applebaum S, Glaspy J, DiCarlo B, Courtney R, Shaik N, Kim S, Fowst C, et al. Phase I study of PD 0332991, a novel, oral, cyclin-D kinase (CDK) 4/6 inhibitor in combination with letrozole, for first-line treatment of metastatic post-menopausal, estrogen receptor-positive (ER+), human epidermal growth factor receptor 2 (HER2)-negative breast cancer. San Antonio: Thirty-Second Annual CTRC‐AACR San Antonio Breast Cancer Symposium; 2009.

[34] Dhillon S (2015). Palbociclib: first global approval. Drugs.

[35] Finn RS, Martin M, Rugo HS, Jones S, Im SA, Gelmon K, Harbeck N, Lipatov ON, Walshe JM, Moulder S (2016). Palbociclib and letrozole in advanced breast cancer. N Engl J Med.

[36] Turner N, André F, M C. Treatment postprogression in women with endocrine-resistant HR+/HER2- advanced breast cancer who received palbociclib plus fulvestrant in PALOMA-3. San Antonio: San Antonio Breast Cancer Symposium (SABCS) 2016; 2016. p. 4-P22.

[37] Walker AJ, Wedam S, Amiri-Kordestani L, Bloomquist E, Tang S, Sridhara R, Chen W, Palmby TR, Fourie ZJ, Fu W (2016). FDA approval of palbociclib in combination with fulvestrant for the treatment of hormone receptor-positive, HER2-negative metastatic breast cancer. Clin Cancer Res.

[38] Cho YS, Angove H, Brain C, Chen CH, Cheng H, Cheng R, Chopra R, Chung K, Congreve M, Dagostin C (2012). Fragment-based discovery of 7-azabenzimidazoles as potent, highly selective, and orally active CDK4/6 inhibitors. Acs Med Chem Lett.

[39] Kim S, Loo A, Chopra R, Caponigro G, Huang A, Vora S, Parasuraman S, Howard S, Keen N, Sellers W, Brain C (2013). Abstract PR02: LEE011: an orally bioavailable, selective small molecule inhibitor of CDK4/6—reactivating Rb in cancer. Mol Cancer Ther.

[40] Rader J, Russell MR, Hart LS, Nakazawa MS, Belcastro LT, Martinez D, Li Y, Carpenter EL, Attiyeh EF, Diskin SJ (2013). Dual CDK4/CDK6 inhibition induces cell-cycle arrest and senescence in neuroblastoma. Clin Cancer Res.

[41] Curigliano G, Gomez PP, Meric-Bernstam F, Conte P, Lolkema MP, Beck JT, Bardia A, Martinez GM, Penault-Llorca F, Dhuria S (2016). Ribociclib plus letrozole in early breast cancer: a presurgical, window-of-opportunity study. Breast.

[42] Hortobagyi GN, Stemmer SM, Burris HA, Yap YS, Sonke GS, Paluch-Shimon S, Campone M, Blackwell KL, Andre F, Winer EP (2016). Ribociclib as first-line therapy for HR-positive, advanced breast cancer. N Engl J Med.

[43] Munster PN, Hamilton EP, Franklin C, Bhansali S, Wan K, Hewes B, Juric D (2014). Phase lb study of LEE011 and BYL719 in combination with letrozole in estrogen receptor-positive, HER2-negative breast cancer (ER+, HER2− BC). J Clin Oncol.

[44] Tolaney SM, Rosen LS, Beeram M, Goldman JW, Gandhi L, Tolcher AW, Papadopoulos KP, Rasco DW, Myrand SP, Kulanthaivel P (2015). Abstract P5-19-13: clinical activity of abemaciclib, an oral cell cycle inhibitor, in metastatic breast cancer. Cancer Res.

[45] Tolaney SM, Beeram M, Beck JT, Conlin AK, Dees EC, Dickler MN, Helsten TL, Conkling PR, Edenfield WJ, Richards DA (2015). A phase Ib study of abemaciclib with therapies for metastatic breast cancer. J Clin Oncol.

[46] Dickler M, Tolaney S, Rugo H, Cortes J, Dieras V, Patt D, Wildiers H, Frenzel M, Koustenis A, Baselga J. MONARCH1: results from a phase II study of abemaciclib, a CDK4 and CDK6 inhibitor, as monotherapy, in patients with HR+/HER2-breast cancer, after chemotherapy for advanced disease. J Clin Oncol. 2016;34(suppl; abstr 510).

[47] Hurvitz S, Martin M, Abad MF, Chan D, Rostorfer R, Petru E, Barriga S, Costigan T, Caldwell C, Nguyen T, et al. Biological effects of abemaciclib in a phase 2 neoadjuvant study for postmenopausal patients with HR+, HER2- breast cancer. In: 2016 SABCS. San Antonio, TX; 2016.

[48] Lu J (2015). Palbociclib: a first-in-class CDK4/CDK6 inhibitor for the treatment of hormone-receptor positive advanced breast cancer. J Hematol Oncol.

[49] Robert C, Long GV, Brady B, Dutriaux C, Maio M, Mortier L, Hassel JC, Rutkowski P, McNeil C, Kalinka-Warzocha E (2015). Nivolumab in previously untreated melanoma without BRAF mutation. N Engl J Med.

[50] Rosenberg JE, Hoffman-Censits J, Powles T, van der Heijden MS, Balar AV, Necchi A, Dawson N, O'Donnell PH, Balmanoukian A, Loriot Y (2016). Atezolizumab in patients with locally advanced and metastatic urothelial carcinoma who have progressed following treatment with platinum-based chemotherapy: a single-arm, multicentre, phase 2 trial. Lancet.

[51] Hodi FS, O'Day SJ, McDermott DF, Weber RW, Sosman JA, Haanen JB, Gonzalez R, Robert C, Schadendorf D, Hassel JC (2010). Improved survival with ipilimumab in patients with metastatic melanoma. N Engl J Med.

[52] Ferris RL, Blumenschein GJ, Fayette J, Guigay J, Colevas AD, Licitra L, Harrington K, Kasper S, Vokes EE, Even C (2016). Nivolumab for recurrent squamous-cell carcinoma of the head and neck. N Engl J Med.

[53] Brahmer J, Reckamp KL, Baas P, Crino L, Eberhardt WE, Poddubskaya E, Antonia S, Pluzanski A, Vokes EE, Holgado E (2015). Nivolumab versus docetaxel in advanced squamous-cell non-small-cell lung cancer. N Engl J Med.

[54] Motzer RJ, Escudier B, McDermott DF, George S, Hammers HJ, Srinivas S, Tykodi SS, Sosman JA, Procopio G, Plimack ER (2015). Nivolumab versus everolimus in advanced renal-cell carcinoma. N Engl J Med.

[55] Garon EB, Rizvi NA, Hui R, Leighl N, Balmanoukian AS, Eder JP, Patnaik A, Aggarwal C, Gubens M, Horn L (2015). Pembrolizumab for the treatment of non-small-cell lung cancer. N Engl J Med.

[56] Brown SD, Warren RL, Gibb EA, Martin SD, Spinelli JJ, Nelson BH, Holt RA (2014). Neo-antigens predicted by tumor genome meta-analysis correlate with increased patient survival. Genome Res.

[57] Loi S, Sirtaine N, Piette F, Salgado R, Viale G, Van Eenoo F, Rouas G, Francis P, Crown JP, Hitre E (2013). Prognostic and predictive value of tumor-infiltrating lymphocytes in a phase III randomized adjuvant breast cancer trial in node-positive breast cancer comparing the addition of docetaxel to doxorubicin with doxorubicin-based chemotherapy: BIG 02-98. J Clin Oncol.

[58] Wimberly H, Brown JR, Schalper K, Haack H, Silver MR, Nixon C, Bossuyt V, Pusztai L, Lannin DR, Rimm DL (2015). PD-L1 expression correlates with tumor-infiltrating lymphocytes and response to neoadjuvant chemotherapy in breast cancer. Cancer Immunol Res.

[59] Ali HR, Glont SE, Blows FM, Provenzano E, Dawson SJ, Liu B, Hiller L, Dunn J, Poole CJ, Bowden S (2015). PD-L1 protein expression in breast cancer is rare, enriched in basal-like tumours and associated with infiltrating lymphocytes. Ann Oncol.

[60] Mittendorf EA, Philips AV, Meric-Bernstam F, Qiao N, Wu Y, Harrington S, Su X, Wang Y, Gonzalez-Angulo AM, Akcakanat A (2014). PD-L1 expression in triple-negative breast cancer. Cancer Immunol Res.

[61] Sabatier R, Finetti P, Mamessier E, Adelaide J, Chaffanet M, Ali HR, Viens P, Caldas C, Birnbaum D, Bertucci F (2015). Prognostic and predictive value of PDL1 expression in breast cancer. Oncotarget.

[62] Ladoire S, Arnould L, Apetoh L, Coudert B, Martin F, Chauffert B, Fumoleau P, Ghiringhelli F (2008). Pathologic complete response to neoadjuvant chemotherapy of breast carcinoma is associated with the disappearance of tumor-infiltrating foxp3+ regulatory T cells. Clin Cancer Res.

[63] Issa-Nummer Y, Darb-Esfahani S, Loibl S, Kunz G, Nekljudova V, Schrader I, Sinn BV, Ulmer HU, Kronenwett R, Just M (2013). Prospective validation of immunological infiltrate for prediction of response to neoadjuvant chemotherapy in HER2-negative breast cancer—a substudy of the neoadjuvant GeparQuinto trial. PLoS One.

[64] Loi S, Michiels S, Salgado R, Sirtaine N, Jose V, Fumagalli D, Kellokumpu-Lehtinen PL, Bono P, Kataja V, Desmedt C (2014). Tumor infiltrating lymphocytes are prognostic in triple negative breast cancer and predictive for trastuzumab benefit in early breast cancer: results from the FinHER trial. Ann Oncol.

[65] Ono M, Tsuda H, Shimizu C, Yamamoto S, Shibata T, Yamamoto H, Hirata T, Yonemori K, Ando M, Tamura K (2012). Tumor-infiltrating lymphocytes are correlated with response to neoadjuvant chemotherapy in triple-negative breast cancer. Breast Cancer Res Treat.

[66] Denkert C, Loibl S, Noske A, Roller M, Muller BM, Komor M, Budczies J, Darb-Esfahani S, Kronenwett R, Hanusch C (2010). Tumor-associated lymphocytes as an independent predictor of response to neoadjuvant chemotherapy in breast cancer. J Clin Oncol.

[67] Mahmoud SM, Paish EC, Powe DG, Macmillan RD, Grainge MJ, Lee AH, Ellis IO, Green AR (2011). Tumor-infiltrating CD8+ lymphocytes predict clinical outcome in breast cancer. J Clin Oncol.

[68] Bates GJ, Fox SB, Han C, Leek RD, Garcia JF, Harris AL, Banham AH (2006). Quantification of regulatory T cells enables the identification of high-risk breast cancer patients and those at risk of late relapse. J Clin Oncol.

[69] Liu S, Lachapelle J, Leung S, Gao D, Foulkes WD, Nielsen TO (2012). CD8+ lymphocyte infiltration is an independent favorable prognostic indicator in basal-like breast cancer. Breast Cancer Res.

[70] Liyanage UK, Moore TT, Joo HG, Tanaka Y, Herrmann V, Doherty G, Drebin JA, Strasberg SM, Eberlein TJ, Goedegebuure PS (2002). Prevalence of regulatory T cells is increased in peripheral blood and tumor microenvironment of patients with pancreas or breast adenocarcinoma. J Immunol.

[71] Muenst S, Schaerli AR, Gao F, Daster S, Trella E, Droeser RA, Muraro MG, Zajac P, Zanetti R, Gillanders WE (2014). Expression of programmed death ligand 1 (PD-L1) is associated with poor prognosis in human breast cancer. Breast Cancer Res Treat.

[72] Ghebeh H, Mohammed S, Al-Omair A, Qattan A, Lehe C, Al-Qudaihi G, Elkum N, Alshabanah M, Bin AS, Tulbah A (2006). The B7-H1 (PD-L1) T lymphocyte-inhibitory molecule is expressed in breast cancer patients with infiltrating ductal carcinoma: correlation with important high-risk prognostic factors. Neoplasia.

[73] Emens LA BFCP. Inhibition of PD-L1 by MPDL3280A leads to clinical activity in patients with metastatic triple-negative breast cancer. In: 2014 San Antonio Breast Cancer Sympsoium. San Antonio, TX; 2014.

[74] Crane CA, Panner A, Murray JC, Wilson SP, Xu H, Chen L, Simko JP, Waldman FM, Pieper RO, Parsa AT (2009). PI(3) kinase is associated with a mechanism of immunoresistance in breast and prostate cancer. Oncogene.

[75] Lehmann BD, Bauer JA, Chen X, Sanders ME, Chakravarthy AB, Shyr Y, Pietenpol JA (2011). Identification of human triple-negative breast cancer subtypes and preclinical models for selection of targeted therapies. J Clin Invest.

[76] Robert C, Schachter J, Long GV, Arance A, Grob JJ, Mortier L, Daud A, Carlino MS, McNeil C, Lotem M (2015). Pembrolizumab versus ipilimumab in advanced melanoma. N Engl J Med.

[77] Langer CJ, Gadgeel SM, Borghaei H, Papadimitrakopoulou VA, Patnaik A, Powell SF, Gentzler RD, Martins RG, Stevenson JP, Jalal SI (2016). Carboplatin and pemetrexed with or without pembrolizumab for advanced, non-squamous non-small-cell lung cancer: a randomised, phase 2 cohort of the open-label KEYNOTE-021 study. Lancet Oncol.

[78] Seiwert TY, Burtness B, Mehra R, Weiss J, Berger R, Eder JP, Heath K, McClanahan T, Lunceford J, Gause C (2016). Safety and clinical activity of pembrolizumab for treatment of recurrent or metastatic squamous cell carcinoma of the head and neck (KEYNOTE-012): an open-label, multicentre, phase 1b trial. Lancet Oncol.

[79] Muro K, Chung HC, Shankaran V, Geva R, Catenacci D, Gupta S, Eder JP, Golan T, Le DT, Burtness B (2016). Pembrolizumab for patients with PD-L1-positive advanced gastric cancer (KEYNOTE-012): a multicentre, open-label, phase 1b trial. Lancet Oncol.

[80] Plimack ER, Bellmunt J, Gupta S, Berger R, Chow LQ, Juco J, Lunceford J, Saraf S, Perini RF, O'Donnell PH (2017). Safety and activity of pembrolizumab in patients with locally advanced or metastatic urothelial cancer (KEYNOTE-012): a non-randomised, open-label, phase 1b study. Lancet Oncol.

[81] Nanda R, Chow LQ, Dees EC, Berger R, Gupta S, Geva R, Pusztai L, Pathiraja K, Aktan G, Cheng JD (2016). Pembrolizumab in patients with advanced triple-negative breast cancer: phase Ib KEYNOTE-012 study. J Clin Oncol.

[82] Rugo H, Delord J, Im S, Ott P, Piha-Paul S, Bedard P, Sachdev J, Le Tourneau C, van Brummelen E, Varga A et al. Preliminary efficacy and safety of pembrolizumab (MK-3475) in patients with PD-L1-positive, estrogen receptor-positive (ER+)/HER2-negative advanced breast cancer enrolled in KEYNOTE-028. In: SABCS 2016. San Antonio, TX; 2015.

[83] Adams S, Diamond J, Hamilton E, Pohlmann P, Tolaney S, Molinero L (2016). Phase Ib trial of atezolizumab in combination with nab-paclitaxel in patients with metastatic triple-negative breast cancer (mTNBC). J Clin Oncol.

[84] Ansell SM, Lesokhin AM, Borrello I, Halwani A, Scott EC, Gutierrez M, Schuster SJ, Millenson MM, Cattry D, Freeman GJ (2015). PD-1 blockade with nivolumab in relapsed or refractory Hodgkin’s lymphoma. N Engl J Med.

[85] Tsai KK, Daud AI (2015). Nivolumab plus ipilimumab in the treatment of advanced melanoma. J Hematol Oncol.

[86] Callahan MK, Bendell JC, Chan E, Morse M, Pillai RN, Bono P, Jaeger D, Evans TRJ, Chau I, Calvo E (2014). Phase I/II, open-label study of nivolumab (anti-PD-1; BMS-936558, ONO-4538) as monotherapy or combined with ipilimumab in advanced or metastatic solid tumors. J Clin Oncol.

[87] Waterhouse D, Gutierrez M, Bekaii-Saab T, DeRosa W, Wainberg Z, George B, Duval Fraser C, Ko A, Pierce D, Stergiopoulos S, Soliman H (2016). Abstract OT1-01-07: nab-paclitaxel (nab-P) plus nivolumab (Nivo) in human epidermal growth factor receptor 2 (HER2)-negative recurrent metastatic breast cancer (MBC). Cancer Res.

[88] Kaufman HL, Russell J, Hamid O, Bhatia S, Terheyden P, D'Angelo SP, Shih KC, Lebbe C, Linette GP, Milella M (2016). Avelumab in patients with chemotherapy-refractory metastatic Merkel cell carcinoma: a multicentre, single-group, open-label, phase 2 trial. Lancet Oncol.

[89] Dirix L, Takacs I, Nikolinakos P, Jerusalem G, Arkenau H-T, Hamilton E, von Heydebreck A, Grote H-J, Chin K, Lippman M (2016). Abstract S1-04: avelumab (MSB0010718C), an anti-PD-L1 antibody, in patients with locally advanced or metastatic breast cancer: a phase Ib JAVELIN solid tumor trial. Cancer Res.

[90] Audebert M, Salles B, Calsou P (2004). Involvement of poly(ADP-ribose) polymerase-1 and XRCC1/DNA ligase III in an alternative route for DNA double-strand breaks rejoining. J Biol Chem.

[91] Dawicki-McKenna JM, Langelier MF, DeNizio JE, Riccio AA, Cao CD, Karch KR, McCauley M, Steffen JD, Black BE, Pascal JM (2015). PARP-1 activation requires local unfolding of an autoinhibitory domain. Mol Cell.

[92] Eustermann S, Wu WF, Langelier MF, Yang JC, Easton LE, Riccio AA, Pascal JM, Neuhaus D (2015). Structural basis of detection and signaling of DNA single-strand breaks by human PARP-1. Mol Cell.

[93] Burkle A (2001). Poly(APD-ribosyl)ation, a DNA damage-driven protein modification and regulator of genomic instability. Cancer Lett.

[94] Farmer H, McCabe N, Lord CJ, Tutt AN, Johnson DA, Richardson TB, Santarosa M, Dillon KJ, Hickson I, Knights C (2005). Targeting the DNA repair defect in BRCA mutant cells as a therapeutic strategy. Nature.

[95] Bryant HE, Schultz N, Thomas HD, Parker KM, Flower D, Lopez E, Kyle S, Meuth M, Curtin NJ, Helleday T (2005). Specific killing of BRCA2-deficient tumours with inhibitors of poly(ADP-ribose) polymerase. Nature.

[96] Turner N, Tutt A, Ashworth A (2005). Targeting the DNA repair defect of BRCA tumours. Curr Opin Pharmacol.

[97] Turner NC, Lord CJ, Iorns E, Brough R, Swift S, Elliott R, Rayter S, Tutt AN, Ashworth A (2008). A synthetic lethal siRNA screen identifying genes mediating sensitivity to a PARP inhibitor. EMBO J.

[98] Matros E, Wang ZC, Lodeiro G, Miron A, Iglehart JD, Richardson AL (2005). BRCA1 promoter methylation in sporadic breast tumors: relationship to gene expression profiles. Breast Cancer Res Treat.

[99] Antoniou A, Pharoah PD, Narod S, Risch HA, Eyfjord JE, Hopper JL, Loman N, Olsson H, Johannsson O, Borg A (2003). Average risks of breast and ovarian cancer associated with BRCA1 or BRCA2 mutations detected in case series unselected for family history: a combined analysis of 22 studies. Am J Hum Genet.

[100] Foulkes WD, Stefansson IM, Chappuis PO, Begin LR, Goffin JR, Wong N, Trudel M, Akslen LA (2003). Germline BRCA1 mutations and a basal epithelial phenotype in breast cancer. J Natl Cancer Inst.

[101] Shah SP, Roth A, Goya R, Oloumi A, Ha G, Zhao Y, Turashvili G, Ding J, Tse K, Haffari G (2012). The clonal and mutational evolution spectrum of primary triple-negative breast cancers. Nature.

[102] Kaufman B, Shapira-Frommer R, Schmutzler RK, Audeh MW, Friedlander M, Balmana J, Mitchell G, Fried G, Stemmer SM, Hubert A (2015). Olaparib monotherapy in patients with advanced cancer and a germline BRCA1/2 mutation. J Clin Oncol.

[103] Kim G, Ison G, McKee AE, Zhang H, Tang S, Gwise T, Sridhara R, Lee E, Tzou A, Philip R (2015). FDA approval summary: olaparib monotherapy in patients with deleterious germline BRCA-mutated advanced ovarian cancer treated with three or more lines of chemotherapy. Clin Cancer Res.

[104] Swisher EM, Lin KK, Oza AM, Scott CL, Giordano H, Sun J, Konecny GE, Coleman RL, Tinker AV, O'Malley DM (2017). Rucaparib in relapsed, platinum-sensitive high-grade ovarian carcinoma (ARIEL2 part 1): an international, multicentre, open-label, phase 2 trial. Lancet Oncol.

[105] Kristeleit R, Shapiro GI, Burris HA, Oza AM, LoRusso PM, Patel M, Domchek SM, Balmana J, Drew Y, Chen LM, et al. A phase I-II study of the oral poly(ADP-ribose) polymerase inhibitor rucaparib in patients with germline BRCA1/2-mutated ovarian carcinoma or other solid tumors. Clin Cancer Res. 2017: clincanres.2796.2016. doi:10.1158/1078-0432. 10.1158/1078-0432.CCR-16-2796 28264872

[106] Mirza MR, Monk BJ, Herrstedt J, Oza AM, Mahner S, Redondo A, Fabbro M, Ledermann JA, Lorusso D, Vergote I (2016). Niraparib maintenance therapy in platinum-sensitive, recurrent ovarian cancer. N Engl J Med.

[107] Tutt A, Robson M, Garber JE, Domchek SM, Audeh MW, Weitzel JN, Friedlander M, Arun B, Loman N, Schmutzler RK (2010). Oral poly(ADP-ribose) polymerase inhibitor olaparib in patients with BRCA1 or BRCA2 mutations and advanced breast cancer: a proof-of-concept trial. Lancet.

[108] Gelmon KA, Tischkowitz M, Mackay H, Swenerton K, Robidoux A, Tonkin K, Hirte H, Huntsman D, Clemons M, Gilks B (2011). Olaparib in patients with recurrent high-grade serous or poorly differentiated ovarian carcinoma or triple-negative breast cancer: a phase 2, multicentre, open-label, non-randomised study. Lancet Oncol.

[109] Lee JM, Peer CJ, Yu M, Amable L, Gordon N, Annunziata CM, Houston N, Goey AK, Sissung TM, Parker B (2017). Sequence-specific pharmacokinetic and pharmacodynamic phase I/Ib study of olaparib tablets and carboplatin in women’s cancer. Clin Cancer Res.

[110] Litton JK, Scoggins M, Ramirez DL, Murthy RK, Whitman GJ, Hess KR, Adrada BE, Moulder SL, Barcenas CH, Valero V (2016). A pilot study of neoadjuvant talazoparib for early-stage breast cancer patients with a BRCA mutation. Ann Oncol.

[111] Osborne CK, Wakeling A, Nicholson RI (2004). Fulvestrant: an oestrogen receptor antagonist with a novel mechanism of action. Br J Cancer.

[112] Wakeling AE, Bowler J (1987). Steroidal pure antioestrogens. J Endocrinol.

[113] Wakeling AE, Dukes M, Bowler J (1991). A potent specific pure antiestrogen with clinical potential. Cancer Res.

[114] Fawell SE, White R, Hoare S, Sydenham M, Page M, Parker MG (1990). Inhibition of estrogen receptor-DNA binding by the “pure” antiestrogen ICI 164,384 appears to be mediated by impaired receptor dimerization. Proc Natl Acad Sci U S A.

[115] Dauvois S, White R, Parker MG (1993). The antiestrogen ICI 182780 disrupts estrogen receptor nucleocytoplasmic shuttling. J Cell Sci.

[116] Nicholson RI, Gee JM, Manning DL, Wakeling AE, Montano MM, Katzenellenbogen BS (1995). Responses to pure antiestrogens (ICI 164384, ICI 182780) in estrogen-sensitive and -resistant experimental and clinical breast cancer. Ann N Y Acad Sci.

[117] Osborne CK, Coronado-Heinsohn EB, Hilsenbeck SG, McCue BL, Wakeling AE, McClelland RA, Manning DL, Nicholson RI (1995). Comparison of the effects of a pure steroidal antiestrogen with those of tamoxifen in a model of human breast cancer. J Natl Cancer Inst.

[118] Wakeling AE (1995). Use of pure antioestrogens to elucidate the mode of action of oestrogens. Biochem Pharmacol.

[119] Wardley AM (2002). Fulvestrant: a review of its development, pre-clinical and clinical data. Int J Clin Pract.

[120] DeFriend DJ, Howell A, Nicholson RI, Anderson E, Dowsett M, Mansel RE, Blamey RW, Bundred NJ, Robertson JF, Saunders C (1994). Investigation of a new pure antiestrogen (ICI 182780) in women with primary breast cancer. Cancer Res.

[121] Singer C, Kuter I, Hegg R, Badwe R, Harbeck N, Bines J, Lowe E (2008). NEWEST: a phase II, randomised, neoadjuvant trial comparing fulvestrant 500 mg vs 250 mg in postmenopausal women with locally advanced, oestrogen receptor-positive (ER+) breast cancer. Eur J Cancer Suppl.

[122] Howell A, Robertson JF, Quaresma AJ, Aschermannova A, Mauriac L, Kleeberg UR, Vergote I, Erikstein B, Webster A, Morris C (2002). Fulvestrant, formerly ICI 182,780, is as effective as anastrozole in postmenopausal women with advanced breast cancer progressing after prior endocrine treatment. J Clin Oncol.

[123] Osborne CK, Pippen J, Jones SE, Parker LM, Ellis M, Come S, Gertler SZ, May JT, Burton G, Dimery I (2002). Double-blind, randomized trial comparing the efficacy and tolerability of fulvestrant versus anastrozole in postmenopausal women with advanced breast cancer progressing on prior endocrine therapy: results of a North American trial. J Clin Oncol.

[124] Chia S, Gradishar W, Mauriac L, Bines J, Amant F, Federico M, Fein L, Romieu G, Buzdar A, Robertson JF (2008). Double-blind, randomized placebo controlled trial of fulvestrant compared with exemestane after prior nonsteroidal aromatase inhibitor therapy in postmenopausal women with hormone receptor-positive, advanced breast cancer: results from EFECT. J Clin Oncol.

[125] Johnston SR, Kilburn LS, Ellis P, Dodwell D, Cameron D, Hayward L, Im YH, Braybrooke JP, Brunt AM, Cheung KL (2013). Fulvestrant plus anastrozole or placebo versus exemestane alone after progression on non-steroidal aromatase inhibitors in postmenopausal patients with hormone-receptor-positive locally advanced or metastatic breast cancer (SoFEA): a composite, multicentre, phase 3 randomised trial. Lancet Oncol.

[126] Bergh J, Jonsson PE, Lidbrink EK, Trudeau M, Eiermann W, Brattstrom D, Lindemann JP, Wiklund F, Henriksson R (2012). FACT: an open-label randomized phase III study of fulvestrant and anastrozole in combination compared with anastrozole alone as first-line therapy for patients with receptor-positive postmenopausal breast cancer. J Clin Oncol.

[127] Mehta RS, Barlow WE, Albain KS, Vandenberg TA, Dakhil SR, Tirumali NR, Lew DL, Hayes DF, Gralow JR, Livingston RB (2012). Combination anastrozole and fulvestrant in metastatic breast cancer. N Engl J Med.

[128] Di Leo A, Jerusalem G, Petruzelka L, Torres R, Bondarenko IN, Khasanov R, Verhoeven D, Pedrini JL, Smirnova I, Lichinitser MR (2010). Results of the CONFIRM phase III trial comparing fulvestrant 250 mg with fulvestrant 500 mg in postmenopausal women with estrogen receptor-positive advanced breast cancer. J Clin Oncol.

[129] Robertson JF, Llombart-Cussac A, Rolski J, Feltl D, Dewar J, Macpherson E, Lindemann J, Ellis MJ (2009). Activity of fulvestrant 500 mg versus anastrozole 1 mg as first-line treatment for advanced breast cancer: results from the FIRST study. J Clin Oncol.

[130] Robertson JF, Bondarenko IM, Trishkina E, Dvorkin M, Panasci L, Manikhas A, Shparyk Y, Cardona-Huerta S, Cheung KL, Philco-Salas MJ (2017). Fulvestrant 500 mg versus anastrozole 1 mg for hormone receptor-positive advanced breast cancer (FALCON): an international, randomised, double-blind, phase 3 trial. Lancet.

[131] Lewis PG, Li G, Dugger DL, Crocker LM, Parsons KL, Mai E, Blattler WA, Lambert JM, Chari RV, Lutz RJ (2008). Targeting HER2-positive breast cancer with trastuzumab-DM1, an antibody-cytotoxic drug conjugate. Cancer Res.

[132] Peddi PF, Hurvitz SA (2013). Trastuzumab emtansine: the first targeted chemotherapy for treatment of breast cancer. Future Oncol.

[133] Baron KB, Brown JR, Heiss BL, Marshall J, Tait N, Tkaczuk KH, Gottlieb SS (2014). Trastuzumab-induced cardiomyopathy: incidence and associated risk factors in an inner-city population. J Card Fail.

[134] Boyraz B, Sendur MA, Aksoy S, Babacan T, Roach EC, Kizilarslanoglu MC, Petekkaya I, Altundag K (2013). Trastuzumab emtansine (T-DM1) for HER2-positive breast cancer. Curr Med Res Opin.

[135] Krop IE, Beeram M, Modi S, Jones SF, Holden SN, Yu W, Girish S, Tibbitts J, Yi JH, Sliwkowski MX (2010). Phase I study of trastuzumab-DM1, an HER2 antibody-drug conjugate, given every 3 weeks to patients with HER2-positive metastatic breast cancer. J Clin Oncol.

[136] Krop IE, LoRusso P, Miller KD, Modi S, Yardley D, Rodriguez G, Guardino E, Lu M, Zheng M, Girish S (2012). A phase II study of trastuzumab emtansine in patients with human epidermal growth factor receptor 2-positive metastatic breast cancer who were previously treated with trastuzumab, lapatinib, an anthracycline, a taxane, and capecitabine. J Clin Oncol.

[137] Perez EA, Barrios C, Eiermann W, Toi M, Im YH, Conte P, Martin M, Pienkowski T, Pivot X, Burris HR (2017). Trastuzumab emtansine with or without pertuzumab versus trastuzumab plus taxane for human epidermal growth factor receptor 2-positive, advanced breast cancer: primary results from the phase III MARIANNE study. J Clin Oncol.

[138] Desai N, Trieu V, Yao Z, Louie L, Ci S, Yang A, Tao C, De T, Beals B, Dykes D (2006). Increased antitumor activity, intratumor paclitaxel concentrations, and endothelial cell transport of cremophor-free, albumin-bound paclitaxel, ABI-007, compared with cremophor-based paclitaxel. Clin Cancer Res.

[139] Gardner ER, Dahut WL, Scripture CD, Jones J, Aragon-Ching JB, Desai N, Hawkins MJ, Sparreboom A, Figg WD (2008). Randomized crossover pharmacokinetic study of solvent-based paclitaxel and nab-paclitaxel. Clin Cancer Res.

[140] Untch M, Jackisch C, Schneeweiss A, Conrad B, Aktas B, Denkert C, Eidtmann H, Wiebringhaus H, Kummel S, Hilfrich J (2016). Nab-paclitaxel versus solvent-based paclitaxel in neoadjuvant chemotherapy for early breast cancer (GeparSepto-GBG 69): a randomised, phase 3 trial. Lancet Oncol.

[141] Gianni L, Mansutti M, Anton A, Calvo L, Bisagni G, Bermejo B (2016). ETNA (evaluating treatment with neoadjuvant abraxane) randomized phase III study comparing neoadjuvant nab-paclitaxel (nab-P) versus paclitaxel (P) both followed by anthracycline regimens in women with HER2-negative high-risk breast cancer: a MICHELANGO study. J Clin Oncol.

[142] Yardley D, Coleman R, Conte P, Cortes J, Brufsky A, Shtivelband M, Young R, Bengala C, Ali H, Eakel J (2017). Abstract P5-15-03: nab-paclitaxel + carboplatin or gemcitabine vs gemcitabine/carboplatin as first-line treatment for patients with triple-negative metastatic breast cancer: results from the randomized phase 2 portion of the tnAcity trial. Cancer Res.

[143] Gradishar WJ, Tjulandin S, Davidson N, Shaw H, Desai N, Bhar P, Hawkins M, O'Shaughnessy J (2005). Phase III trial of nanoparticle albumin-bound paclitaxel compared with polyethylated castor oil-based paclitaxel in women with breast cancer. J Clin Oncol.

[144] Brufsky A (2017). Nab-paclitaxel for the treatment of breast cancer: an update across treatment settings. Exp Hematol Oncol.

[145] Sylvia A. Phase Ib trial of atezolizumab in combination with nab-paclitaxel in patients with metastatic triple-negative breast cancer (mTNBC). In: 2016 ASCO Annual meeting. Chicago, IL; 2016.

[146] Hirata, Uemura Y (1986). Halichondrins—antitumor polyether macrolides from a marine sponge. Pure Appl Chem.

[147] Pettit GR, Herald CL, Boyd MR, Leet JE, Dufresne C, Doubek DL, Schmidt JM, Cerny RL, Hooper JN, Rutzler KC (1991). Isolation and structure of the cell growth inhibitory constituents from the western Pacific marine sponge Axinella sp. J Med Chem.

[148] Jordan MA, Kamath K, Manna T, Okouneva T, Miller HP, Davis C, Littlefield BA, Wilson L (2005). The primary antimitotic mechanism of action of the synthetic halichondrin E7389 is suppression of microtubule growth. Mol Cancer Ther.

[149] Yoshida T, Ozawa Y, Kimura T, Sato Y, Kuznetsov G, Xu S, Uesugi M, Agoulnik S, Taylor N, Funahashi Y (2014). Eribulin mesilate suppresses experimental metastasis of breast cancer cells by reversing phenotype from epithelial-mesenchymal transition (EMT) to mesenchymal-epithelial transition (MET) states. Br J Cancer.

[150] Agoulnik SI, Kawano S, Taylor N, Oestreicher J, Matsui J, Chow J, Oda Y, Funahashi Y (2014). Eribulin mesylate exerts specific gene expression changes in pericytes and shortens pericyte-driven capillary network in vitro. Vasc Cell.

[151] Funahashi Y, Okamoto K, Adachi Y, Semba T, Uesugi M, Ozawa Y, Tohyama O, Uehara T, Kimura T, Watanabe H (2014). Eribulin mesylate reduces tumor microenvironment abnormality by vascular remodeling in preclinical human breast cancer models. Cancer Sci.

[152] Cortes J, O'Shaughnessy J, Loesch D, Blum JL, Vahdat LT, Petrakova K, Chollet P, Manikas A, Dieras V, Delozier T (2011). Eribulin monotherapy versus treatment of physician’s choice in patients with metastatic breast cancer (EMBRACE): a phase 3 open-label randomised study. Lancet.

[153] Kaufman PA, Awada A, Twelves C, Yelle L, Perez EA, Velikova G, Olivo MS, He Y, Dutcus CE, Cortes J (2015). Phase III open-label randomized study of eribulin mesylate versus capecitabine in patients with locally advanced or metastatic breast cancer previously treated with an anthracycline and a taxane. J Clin Oncol.

[154] Twelves C, Cortes J, Vahdat L, Olivo M, He Y, Kaufman PA, Awada A (2014). Efficacy of eribulin in women with metastatic breast cancer: a pooled analysis of two phase 3 studies. Breast Cancer Res Treat.

[155] Tolaney S, Savulsky C, Aktan G, Xing D, Almonte A, Karantza V, Diab S. Phase 1b/2 study to evaluate eribulin mesylate in combination with pembrolizumab in patients with metastatic triple-negative breast cancer. In: SABCS 2016. San Antonio, TX; 2016.

[156] Giannakakou P, Gussio R, Nogales E, Downing KH, Zaharevitz D, Bollbuck B, Poy G, Sackett D, Nicolaou KC, Fojo T (2000). A common pharmacophore for epothilone and taxanes: molecular basis for drug resistance conferred by tubulin mutations in human cancer cells. Proc Natl Acad Sci U S A.

[157] Zhang P, Sun T, Zhang Q, Yuan Z, Jiang Z, Wang XJ, Cui S, Teng Y, Hu XC, Yang J (2017). Utidelone plus capecitabine versus capecitabine alone for heavily pretreated metastatic breast cancer refractory to anthracyclines and taxanes: a multicentre, open-label, superiority, phase 3, randomised controlled trial. Lancet Oncol.

[158] Dai H, Wang Y, Lu X, Han W. Chimeric antigen receptors modified T-cells for cancer therapy. J Natl Cancer Inst. 2016;108(7). 10.1093/jnci/djv439 PMC4948566 26819347

[159] Maude S, Barrett DM (2016). Current status of chimeric antigen receptor therapy for haematological malignancies. Br J Haematol.

[160] Wang Z, Wu Z, Liu Y, Han W (2017). New development in CAR-T cell therapy. J Hematol Oncol.

[161] Necela BM, Crozier JA, Andorfer CA, Lewis-Tuffin L, Kachergus JM, Geiger XJ, Kalari KR, Serie DJ, Sun Z, Moreno-Aspitia A (2015). Folate receptor-alpha (FOLR1) expression and function in triple negative tumors. PLoS One.

[162] Hartmann LC, Keeney GL, Lingle WL, Christianson TJ, Varghese B, Hillman D, Oberg AL, Low PS (2007). Folate receptor overexpression is associated with poor outcome in breast cancer. Int J Cancer.

[163] Song DG, Ye Q, Poussin M, Harms GM, Figini M, Powell DJ (2012). CD27 costimulation augments the survival and antitumor activity of redirected human T cells in vivo. Blood.

[164] Song DG, Ye Q, Carpenito C, Poussin M, Wang LP, Ji C, Figini M, June CH, Coukos G, Powell DJ (2011). In vivo persistence, tumor localization, and antitumor activity of CAR-engineered T cells is enhanced by costimulatory signaling through CD137 (4-1BB). Cancer Res.

[165] Song DG, Ye Q, Poussin M, Chacon JA, Figini M, Powell DJ (2016). Effective adoptive immunotherapy of triple-negative breast cancer by folate receptor-alpha redirected CAR T cells is influenced by surface antigen expression level. J Hematol Oncol.

[166] Goto Y, Kurozumi A, Enokida H, Ichikawa T, Seki N (2015). Functional significance of aberrantly expressed microRNAs in prostate cancer. Int J Urol.

[167] Lowery AJ, Miller N, Devaney A, McNeill RE, Davoren PA, Lemetre C, Benes V, Schmidt S, Blake J, Ball G (2009). MicroRNA signatures predict oestrogen receptor, progesterone receptor and HER2/neu receptor status in breast cancer. Breast Cancer Res.

[168] Crippa E, Lusa L, De Cecco L, Marchesi E, Calin GA, Radice P, Manoukian S, Peissel B, Daidone MG, Gariboldi M (2014). miR-342 regulates BRCA1 expression through modulation of ID4 in breast cancer. PLoS One.

[169] He YJ, Wu JZ, Ji MH, Ma T, Qiao EQ, Ma R, Tang JH (2013). miR-342 is associated with estrogen receptor-alpha expression and response to tamoxifen in breast cancer. Exp Ther Med.

[170] Fkih MI, Privat M, Ponelle F, Penault-Llorca F, Kenani A, Bignon YJ (2015). Identification of miR-10b, miR-26a, miR-146a and miR-153 as potential triple-negative breast cancer biomarkers. Cell Oncol.

[171] Zhao JJ, Lin J, Yang H, Kong W, He L, Ma X, Coppola D, Cheng JQ (2008). MicroRNA-221/222 negatively regulates estrogen receptor alpha and is associated with tamoxifen resistance in breast cancer. J Biol Chem.

[172] Gan R, Yang Y, Yang X, Zhao L, Lu J, Meng QH (2014). Downregulation of miR-221/222 enhances sensitivity of breast cancer cells to tamoxifen through upregulation of TIMP3. Cancer Gene Ther.

[173] Masri S, Liu Z, Phung S, Wang E, Yuan YC, Chen S (2010). The role of microRNA-128a in regulating TGFbeta signaling in letrozole-resistant breast cancer cells. Breast Cancer Res Treat.

[174] Jung EJ, Santarpia L, Kim J, Esteva FJ, Moretti E, Buzdar AU, Di Leo A, Le XF, Bast RJ, Park ST (2012). Plasma microRNA 210 levels correlate with sensitivity to trastuzumab and tumor presence in breast cancer patients. Cancer.

[175] Moskwa P, Buffa FM, Pan Y, Panchakshari R, Gottipati P, Muschel RJ, Beech J, Kulshrestha R, Abdelmohsen K, Weinstock DM (2011). miR-182-mediated downregulation of BRCA1 impacts DNA repair and sensitivity to PARP inhibitors. Mol Cell.

[176] Iliopoulos D, Kavousanaki M, Ioannou M, Boumpas D, Verginis P (2011). The negative costimulatory molecule PD-1 modulates the balance between immunity and tolerance via miR-21. Eur J Immunol.

[177] Naidu S, Magee P, Garofalo M (2015). MiRNA-based therapeutic intervention of cancer. J Hematol Oncol.

